# Body mass index is similar to alternative anthropometric indices in evaluating plasma lipids as proxy for cardiovascular disease in women with previous hypertensive disorders of pregnancy: A cross-sectional study

**DOI:** 10.1177/17455057241310316

**Published:** 2025-01-28

**Authors:** Kristina Klepp, Anne Cathrine Staff, Meryam Sugulle, Kjartan Moe

**Affiliations:** 1 Institute of Clinical Medicine, Faculty of Medicine, University of Oslo, Oslo, Norway; 2 Division of Gynaecology and Obstetrics, Ullevål, Oslo University Hospital, Oslo, Norway; 3 Department of Obstetrics and Gynaecology, Bærum Hospital, Vestre Viken Hospital Trust, Drammen, Norway

**Keywords:** hypertension in pregnancy, preeclampsia, obesity, obstetrics, women’s health

## Abstract

**Background:**

Women with previous hypertensive disorders of pregnancy (HDP) have increased risk of cardiovascular disease (CVD). Overweight is a modifiable risk factor for both conditions. Anthropometric indices such as waist circumference, hip circumference, waist-to-hip ratio, estimated total body fat, a body shape index, waist-to-hip-to-height ratio, and index of central obesity improve estimation of cardiovascular death risk in the general population as compared to body mass index (BMI).

**Objectives:**

We aimed to assess whether alternative body mass composition indices associate more strongly with postpartum blood lipid levels, as a proxy for CVD risk, than BMI. We also aimed to investigate whether associations differ between women with previous normotensive or hypertensive index pregnancies.

**Design:**

In this cross-sectional study, we examined 296 women 1 or 3 years after an index pregnancy that was normotensive (*n* = 116) or complicated by a hypertensive pregnancy disorder, including preeclampsia (*n* = 133) or gestational hypertension (*n* = 47).

**Methods:**

Uni- and multivariable regression analyses, adjusted for age and smoking, were conducted to evaluate associations between postpartum body mass composition indices and blood lipids. *p* < 0.05 was considered statistically significant.

**Results:**

Median BMI and overweight rates were higher in women with previous HDP than in controls (23.9 kg/m^2^ versus 22.8 kg/m^2^ and 44.4% versus 30.2%, both *p* ⩽ 0.03). No body mass composition indices in any pregnancy complication group showed stronger associations with adverse lipid levels than BMI. However, women with previous HDP more often displayed significant associations between adverse body mass composition indices and adverse lipid levels, compared with controls.

**Conclusion:**

Alternative anthropometric measurements are not better suited to evaluate circulating lipids as proxy for CVD risk after HDP, compared to BMI. We hence recommend using BMI in CVD risk assessment after HDP due to its current widespread use and feasibility.

## Plain language summary

Cardiovascular disease risk evaluation in women after hypertensive pregnancy disorders: A study of the role of traditional and nontraditional body mass composition indices

*Why was the study done*?

Women who have experienced hypertensive disorders of pregnancy have increased risk of developing cardiovascular disease later in life. Overweight is a well-known risk factor for both cardiovascular disease and hypertensive disorders of pregnancy. Body mass index (BMI) is the most common used tool to evaluate overweight, but other indices to evaluate body mass composition may be better suited to evaluate cardiovascular death risk in the general population. Lipids are among several modifiable risk factors for cardiovascular disease. We therefore aimed to investigate associations between alternative body mass composition indices and risk of cardiovascular disease, using blood lipids as a marker for cardiovascular disease, in women with previous hypertensive disorders of pregnancy and women with previous uncomplicated pregnancies.

*What did the researchers do*?

We examined 296 women one or three years after pregnancy. Our study group included 180 women with previous hypertensive pregnancy disorder and 116 women with previous uncomplicated pregnancies as controls. We analyzed associations between different body mass composition indices and blood lipid levels, to evaluate the risk of cardiovascular disease after pregnancy.

*What did the researchers find*?

Women with previous hypertensive pregnancy disorders were more often overweight, compared with women with previous uncomplicated pregnancies, and showed in general higher BMIs. We found no differences between the different body mass composition indices and their associations with blood lipids. Women with previous hypertensive pregnancy disorders did however display more frequent associations between body mass composition indices and circulating lipids, compared with the control group.

*What do the findings mean*?

Following hypertensive disorders of pregnancy, women more often have elevated BMI. Their BMI is comparable to alternative body mass composition indices in association with circulating lipids. This supports using BMI in the assessment of cardiovascular disease risk in this group at high risk of cardiovascular disease development.

## Graphical abstract

**Graphical abstract:**
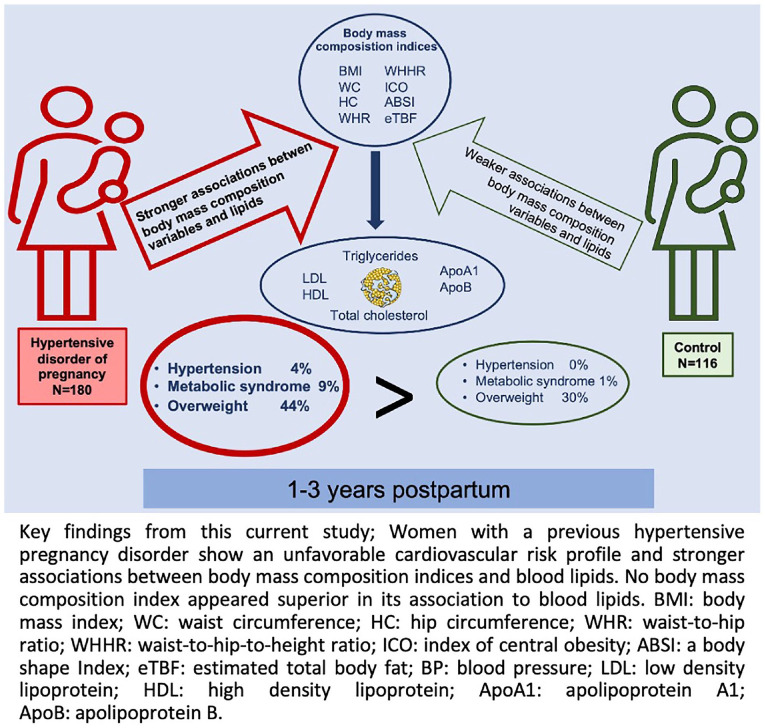

## Introduction

Cardiovascular disease (CVD) is the leading cause of death worldwide.^1^ Women with previous hypertensive disorders of pregnancy (HDP) have a two- to eight-fold increased CVD risk.^2^ Women with previous HDP show an excess risk of hypertension within 10 years after pregnancy.^3^ Important risk factors for both CVD and HDP, in addition to age, include obesity, dyslipidemia, physical inactivity, insulin resistance, malnutrition, and psychosocial factors,^4–6^ many of which are modifiable.^6^

Obesity is associated with an increased CVD risk,^7^ as well as an increased risk of all-cause mortality.^8^ Body mass index (BMI) is a simple and commonly used tool to evaluate underweight, overweight, and obesity.^9^ Nevertheless, its usefulness has been debated due to its age and sex dependency.^10^ BMI neither distinguishes between lean body mass and fat mass nor provides information about fat distribution.^11^ This is a limitation, as abdominal obesity is a stronger risk factor for CVD than generalized obesity.^5,9,12^

Waist circumference (WC), hip circumference (HC), and waist-to-hip ratio (WHR) are other traditionally used body mass composition indices, improving CVD risk prediction among middle-aged and older persons as compared to BMI.^13–15^ Even normal-weight women with elevated WC have increased CVD risk.^16^ Nontraditional adiposity indices seem to associate more strongly with cardiovascular mortality than BMI in the general population.^17^ These indices include estimated total body fat (eTBF), a body shape index (ABSI), and waist-to-height ratio, the latter also named index of central obesity (ICO). Also, waist-to-hip-to-height ratio (WHHR) is found to be a stronger predictor for cardiovascular mortality than BMI.^18^ Although most studies agree that BMI is inferior to other anthropometric indices in CVD risk prediction,^13,18–21^ there is no clear consensus regarding the optimal measure of abdominal obesity in predicting CVD risk.^9^

It is important to establish appropriate screening tools for assessing CVD risk at a young age, where preventive strategies likely are most efficient. We recently showed that current CVD risk scores are unhelpful in stratifying CVD risk in young women after pregnancy complications.^22^ Women are underrepresented in clinical CVD trials,^23^ and to our knowledge publications of associations between body mass composition indices and modifiable CV risk factors after HDP are lacking. We therefore aimed to investigate whether alternative body mass composition indices associate more strongly with postpartum dyslipidemia, as a proxy for cardiovascular risk, than traditional indices, 1- or 3-years postpartum. Additionally, we aimed to evaluate whether associations differed between women with a previous HDP, compared with normotensive controls.

## Methods

### Study population

Participants in this cross-sectional HAPPY (health after pregnancy complications) study (performed in 2013–2019) were women ⩾18 years who delivered a singleton baby at Oslo University Hospital, Ullevål, Norway, 1 or 3 years previously. All participants received written and oral information, as previously described.^24,25^ The present substudy included all women from the HAPPY study with an index pregnancy complicated by HDP or an uncomplicated (normotensive and euglycemic) index pregnancy, with body composition and circulating lipid data available. General exclusion criteria included pregestational or gestational diabetes, rheumatic or renal disease, ongoing malignant disease, any chronic CVD (except for HDP), and ongoing pregnancy or breastfeeding. For women who participated both 1 and 3 years postpartum, 3-year data were analyzed. For women pregnant between 1- and 3-year examinations, 1-year data were analyzed. Thus, we analyzed 1-year data from 205 women (69.3%) and 3-year data from 91 women (30.7%) (Supplemental Figure S1 and Supplemental Table S1).

This manuscript is prepared following the STROBE guidelines.^26^ For the scope of this paper, the term “woman/women” is used to refer to a person’s sex relating to people who have been pregnant. Furthermore, the term “woman” is intended to presume neither gender identity nor expression.

### Operational definitions

The HDP group encompassed index pregnancies complicated by preeclampsia (PE), gestational hypertension (GH), or chronic hypertension with superimposed PE. GH was defined as new-onset hypertension (systolic blood pressure (SBP) ⩾140 mmHg and/or diastolic BP (DBP) ⩾90 mmHg) without proteinuria or any evidence of hematological or biochemical complications after gestational week 20.^27^ PE was defined as GH and at least one new-onset PE-associated feature after gestational week 20 (e.g., new-onset proteinuria, elevated transaminases, small-for-gestational-age (SGA) or stillbirth).^27^ Chronic hypertension with superimposed PE was defined as hypertension predating the pregnancy, or diagnosed before gestational week 20, and at least one new-onset PE-associated feature after gestational week 20. Women with eclampsia and/or hemolysis, elevated liver enzymes and low platelets (HELLP) or partial HELLP in their index pregnancy were also classified as having PE. SGA was defined by newborns (without chromosomal abnormalities) with weight below the third percentile, adjusted for fetal sex and gestational age at delivery.^28^

The control group included women with normotensive and euglycemic index pregnancies, delivering at term (⩾gestational week 37^0^), without PE in any previous pregnancy. Women who delivered SGA newborns were excluded from the control group (Supplemental Figure S1, Flowchart).

### Clinical cardiovascular assessments and blood sampling

In-patient hospital BP was measured according to clinical routines with a validated device (Dinamap Pro, 100VE; GE Medical Systems Information Technology, Inc., Milwaukee, WI, USA). At the 1- and 3-year follow-up examinations, BP was measured in the right upper arm, after 10 min rest in supine position, with the head slightly elevated, with an identical BP device as prior to delivery. A supine position was chosen to save time for the patient, as BP measurement was followed by a clinical examination that needed prior bed rest. BP was measured with three consecutive measurements, and mean BP was calculated based on the two last BPs.

Fasting (⩾6 h) morning serum samples were collected at the follow-up examination.^24^ Lipid and blood glucose analyses of the HAPPY study are previously presented for another HAPPY subgroup^24^ and included total cholesterol, triglycerides, low-density lipoprotein (LDL) cholesterol, high-density lipoprotein (HDL) cholesterol, apolipoprotein A1 (ApoA1), apolipoprotein B (ApoB), glucose, and HbA1c. Serum glucose 2 h after oral glucose tolerance test (OGTT) (75 g glucose in 250–300 mL water) was also measured. Age- and sex-specific reference values are displayed in Supplemental Table S2. Dyslipidemia was defined as total cholesterol, LDL cholesterol, triglycerides and lipoprotein ApoB values above reference values, and HDL cholesterol and lipoprotein A1 below reference values.^29^ Metabolic syndrome was defined according to the NCEP ATP III definition.^30^ Participants also completed questionnaires regarding previous pregnancies (before index pregnancy), cardiovascular health, traditional CV risk factors, general health, and socioeconomic factors.

### Body mass composition indices

Anthropometric and clinical measurements at follow-up examinations were conducted using standardized methods. Weight and height were measured with the participant in light clothing, without shoes. Women were classified at follow-up as normal weight, overweight, or obese according to the World Health Organization; BMI 18.5–24.9, ⩾25.0–29.9 or BMI ⩾30.0 kg/m^2^, respectively.^31^

WC was measured at the end of a normal expiration, as the horizontal circumference midway between the lowest rib and the iliac crest, and HC measured horizontally at the widest part of the hip. Both were conducted with a non-stretchable band, to the nearest centimeters, with the study participant standing with the arms hanging down.^9^

Supplemental Table S3 depicts all body composition indices used in the present study. WHR was calculated as WC divided by HC.^9^ ICO was calculated as WC (cm) divided by height (cm).^15,32^ WHHR was calculated as WC (m) divided by HC (m) divided by height (m).^18^ eTBF was calculated by the sex-specific Young Man’s Christian Association (YMCA) formula for females as: 100 ×  (−76.76 +  [4.15 ×  WC (in.)] −  [0.082 × weight (lbs)])/weight),^33^ and ABSI was calculated as: WC [m]/(BMI^2/3^ × height^1/2^ [m]).^34^

### Statistical analyses

Statistical analyses were performed with the Statistical Package for the Social Sciences (PASW Statistics 26 and 28) and statistical software for data science (Stata Statistical Software: Release 17. College Station, TX, STATACorp LLC). Due to the secondary nature of this study, analyses have been conducted for all patients with relevant data available. No sample size analyses have been performed. Results are presented as medians (and range), as most continuous variables were not normally distributed. The Mann–Whitney *U* test was used to test differences between groups. Categorical variables are presented as rate (%), and differences between groups tested by Fisher’s mid-*p* corrected test. Uni- and multivariable regression analyses, adjusted for classical cardiovascular risk factors, were conducted to evaluate associations between adiposity indices and blood lipids. Unadjusted and adjusted spline regression analyses were conducted for all study groups to test linearity before conducting linear regression (exemplified for the total cohort in Supplemental Figures S2A–S9F). Missing data were analyzed as missing for all variables, and no imputations were made. A *p*-value <0.05 was considered statistically significant.

## Results

We included 452 women in the HAPPY study, 1 or 3 years postpartum (Supplemental Figure S1). Among these, we selected women with a relevant index pregnancy diagnosis, and available body mass composition and blood lipid data. The remaining 296 women had a euglycemic index pregnancy complicated either by a hypertensive disorder (HDP: *n* = 180) or an uncomplicated normotensive pregnancy (*n* = 116). The HDP group consisted of 133 women with previous PE and 47 with previous GH.

Median age at follow-up was 35 years in the total cohort and was not significantly different between study groups. Women with previous PE had significantly higher BMI than controls (24.0 kg/m^2^ versus 22.8 kg/m^2^, *p* = 0.03), as well as significantly higher overweight rate (BMI ⩾25 kg/m^2^) (45.1% versus 30.2%, *p* = 0.02). Women with previous PE showed significantly higher HC (102 cm vs 100 cm, *p* = 0.04), and significantly lower ABSI (0.072 m^11/6^ kg^−2/3^ versus 0.074 m^11/6^ kg^−2/3^, *p* < 0.01), both compared with controls. Other body mass composition indices were not significantly different between study groups. In the total cohort, most women were of white ethnicity (*n* = 245; 82.8%) and had higher education (>high school: *n* = 207; 69.9%), and few women (*n* = 24; 8.1%) were smokers at postpartum follow-up. There were no statistically significant differences between study groups concerning these parameters, apart from significantly higher rate of white ethnicity in the GH group, compared with controls (93.6% versus 79.3%, respectively, *p* = 0.03). Clinical group characteristics at follow-up are shown in Table 1 and Supplemental Table S1.

**Table 1.** Clinical characteristics of the study population, 1 or 3 years postpartum (*n* = 296), by index pregnancy group. Data are presented as medians (range) for continuous variables, and as rates (%) for categorical variables. *p*-Values are compared with those of controls. *p*-Value <0.05 are marked with asterix (*).

Characteristic	Total (*n* = 296)	Controls (*n* = 116)	HDP (*n* = 180)	PE (*n* = 133)	GH (*n* = 47)	Total missing (*n*)
Age (years)	35 (23–48)	36 (24–48)	35 (23–47)	35 (24–47)	35 (23–47)	0
SBP (mmHg)	111 (90–160)	106 (90–127)	115 (92–160)*	114 (92–160)*	119 (99–145)*	0
DBP (mmHg)	67 (51–96)	63 (52–83)	71 (51–96)*	70 (51–92)*	74 (53–96)*	0
Hypertension (SBP ⩾140 or DBP ⩾90)	2.7% (8)	0.0% (0)	4.4% (8)*	3.8% (5)*	6.4% (3)*	0
Stage 1 hypertension (SBP ⩾ 130 < 140 or DBP ⩾ 80 < 90)	10.1% (30)	1.7% (2)	15.6% (28)*	12.0% (16)*	25.5% (12)*	0
Anthropometric data						
Weight (kg)	65 (45–118)	64 (47–117)	67 (45–118)	67 (45–118)	67 (48–102)	0
Height (cm)	168 (150–185)	168 (153–185)	168 (150–182)	167 (150–182)	168 (156–182)	0
BMI (kg/m^2^)	23.5 (17.1–39.5)	22.8 (17.1–36.0)	23.9 (18.1–39.5)*	24.0 (18.3–39.5)*	23.4 (18.1–35.5)	0
Overweight (BMI ⩾25 kg/m^2^)	38.9% (115)	30.2% (35)	44.4% (80)*	45.1% (60)*	42.6% (20)	0
WC (cm)	82 (64–132)	81 (64–132)	82 (68–119)	82 (68–119)	84 (70–106)	0
HC (cm)	101 (72–132)	100 (72–127)	101 (85–132)	102 (85–132)*	100 (89–123)	0
WHR	0.77 (0.64–1.11)	0.77 (0.64–1.11)	0.76 (0.67–1.07)	0.76 (0.67–1.07)	0.77 (0.69–0.89)	0
WHHR (m^−1^)	0.46 (0.37–0.66)	0.46 (0.38–0.66)	0.46 (0.37–0.64)	0.45 (0.37–0.64)	0.46 (0.38–0.56)	0
ICO (WHtR)	0.46 (0.36–0.70)	0.46 (0.36–0.69)	0.46 (0.39–0.70)	0.46 (0.39–0.70)	0.46 (0.39–0.62)	0
ABSI (m^11/6^ kg^−2/3^)	0.073 (0.063–0.086)	0.074 (0.065–0.085)	0.072 (0.063–0.086)*	0.072 (0.063–0.086)*	0.073 (0.067–0.083)	0
eTBF (%)	25.2 (13.8–46.2)	25.2 (13.8–45.2)	25.2 (17.0–46.2)	24.9 (17.0–46.2)	26.3 (17.7–37.2)	0
Circulating biomarkers						
LDL cholesterol (mmol/L)	2.73 (1.10–4.86)	2.70 (1.53–4.70)	2.73 (1.10–4.86)	2.77 (1.15–4.86)	2.58 (1.10–4.03)	0
HDL cholesterol (mmol/L)	1.53 (0.71–3.03)	1.55 (1.00–2.93)	1.51 (0.71–3.03)	1.50 (0.71–3.03)	1.51 (0.80–2.60)	0
ApoA1 (g/L)	1.46 (1.01–2.48)	1.48 (1.04–2.48)	1.44 (1.01–2.36)	1.44 (1.01–2.34)	1.44 (1.04–2.36)	6
ApoB (g/L)	0.82 (0.37–1.62)	0.81 (0.51–1.62)	0.83 (0.37–1.60)	0.84 (0.37–1.60)	0.81 (0.42–1.20)	4
Cholesterol (mmol/L)	4.40 (2.70–7.20)	4.40 (2.70–6.30)	4.50 (2.80–7.20)	4.40 (3.00–7.20)	4.50 (2.80–6.20)	0
Triglycerides (mmol/L)	0.70 (0.21–3.11)	0.67 (0.30–1.83)	0.72 (0.21–3.11)	0.73 (0.21–3.11)	0.70 (0.21 – 2.25)	0

The Mann–Whitney *U* test for continuous variables and Fisher’s mid-*p* corrected test for categorical variables comparing pregnancy complication groups (e.g., preeclampsia) to controls. Values presented as medians (ranges) or rates (*n*). PE: preeclampsia; GH: gestational hypertension; HDP: hypertensive disorders of pregnancy (including both PE and GH patients); BMI: body mass index; WC: waist circumference; HC: hip circumference; WHR: waist-to-hip ratio; WHHR: waist-to-hip-to-height ratio; ICO: index of central obesity; ABSI: a body shape index; eTBF: estimated total body fat; SBP: systolic blood pressure; DBP: diastolic blood pressure; LDL: low-density lipoprotein; HDL: high-density lipoprotein; ApoA1: apolipoprotein A1; ApoB: apolipoprotein B; CVD: cardiovascular disease; WHtR: waist-to-height ratio.

Both median systolic and diastolic blood pressures were significantly higher at follow-up for the previous PE and GH groups than for controls (SBP; 114 and 119 mmHg, respectively, versus 106 mmHg and DBP; 70 and 74 mmHg, respectively, versus 63 mmHg, all *p* < 0.01). Only 8 (2.7%) women in the total cohort were hypertensive at follow-up (SBP ⩾140 and/or DBP ⩾90 mmHg), all belonging to the previous HDP group (5 women with previous PE and 3 with previous GH) (Table 1).

Detailed data from the index pregnancy are provided in Supplemental Table S4. Briefly, women in the HDP group had significantly higher prepregnancy BMI (23.4 kg/m^2^ versus 22.4 kg/m^2^, *p* = 0.02), were significantly more often primiparous (70.0% versus 37.9%, *p* < 0.01), had significantly lower gestational age at delivery (week 38.0 versus 39.3, *p* < 0.01) and lower offspring birthweight (2844 g versus 3543 g, *p* < 0.01), all compared with controls.

Women in our cohort were generally metabolically healthy, as indicated by the biomarker levels (Table 1). No patients tested positive for diabetes on the OGTT,^29^ but two women in the previous PE group (1.5%) showed impaired glucose tolerance. Also, blood lipids displayed no significant differences between study groups. One or more dyslipidemic values were present in 24 women (8.1%) of the total cohort (Supplemental Table S5). No woman showed abnormal values for all blood lipids, but eight women showed more than one dyslipidemic value. The previous PE group displayed highest dyslipidemia rate (13 women; 9.8%), followed by the GH group (4 women; 8.5%). The dyslipidemia rate was higher in women with previous HDP (*n* = 17; 9.4%) than in the control group (*n* = 7; 6.0%), although not statistically significant (*p* = 0.29).

In the total study population, 17 women (5.7%) fulfilled the metabolic syndrome criteria.^30^ Only 1 of these women belonged to the control group, while 16 had HDP in their index pregnancy (PE; *n* = 13 or GH; *n* = 3). The higher rate of metabolic syndrome in the previous PE group (9.8%) was statistically significant compared with controls (*p* < 0.01).

Spline regression analyses showed acceptable linear relationships between all body composition indices and lipid values, in all study groups (exemplified in Supplemental Figures S2A–S9F), supporting our use of linear regression methods.

In the total cohort, univariable regression analyses showed significant associations between most body composition indices and lipid values (Supplemental Table S6). The conclusions remained mostly the same following multivariable regression analyses, adjusted for age and smoking (Supplemental Table S7) and sensitivity analyses for 1- and 3-year data (data not shown).

In univariable regression analyses for the control group, most body mass composition indices showed significant associations with circulating lipid values (Supplemental Table S8), but multivariable regression analyses showed few significant associations (Table 2, beta values and confidence intervals are displayed in Supplemental Table S9). Multivariable analyses showed significant associations between WC and all blood lipids, except from ApoA1. BMI, ICO, and eTBF also showed significant associations with most blood lipids, while WHR, WHHR, and ABSI only showed significant associations with triglycerides. HC showed significant associations with only LDL cholesterol and triglycerides (*R*^2^ = 10.2, *p* = 0.04 and *R*^2^ = 8.1, *p* < 0.01, respectively). No body mass composition index showed significant associations with ApoA1. The pattern of associations with lipid values varied between body mass composition indices, but no body mass composition index appeared more closely associated with any lipid category values compared with others.

**Table 2.** Associations between body mass composition indices and blood lipids for the control group at follow-up (*n* = 116). Significant associations (*p* < 0.05) are marked with asterix (*).

Control								
	BMI	WC	HC	WHR	WHHR	ICO	ABSI	eTBF
	*R*^2^ (%)	*R*^2^ (%)	*R*^2^ (%)	*R*^2^ (%)	*R*^2^ (%)	*R*^2^ (%)	*R*^2^ (%)	*R*^2^ (%)
LDL	11.94*	13.70*	10.15*	9.42	7.84	12.02*	7.47	10.63*
HDL	6.54*	6.97*	3.82	4.34	3.87	7.12*	2.15	6.92*
Total cholesterol	9.18	10.50*	7.66	8.71	7.78	9.79	7.39	9.01
Triglycerides	18.87*	26.26*	8.12*	13.93*	10.67*	22.71*	7.16*	16.42*
ApoA1	2.33	1.76	1.98	1.65	1.72	2.16	1.45	2.16
ApoB	8.59	10.52*	7.03	9.46	8.79	9.94*	8.02	10.63*

*R*^2^ values for multivariable regression analyses. BMI: body mass index; WC: waist circumference; HC: hip circumference; WHR: waist-to-hip ratio; WHHR: waist-to-hip-to-height ratio; ICO: index of central obesity; ABSI: a body shape index; eTBF: estimated total body fat; LDL: low-density lipoprotein; HDL: high-density lipoprotein; ApoA1: apolipoprotein A1; ApoB: apolipoprotein B.

In the HDP group, most body mass composition indices showed significant associations with blood lipids in univariable regression analyses (Supplemental Table S10), and conclusions were mostly the same following multivariable regression analyses (Supplemental Table S11). No body mass composition index was more closely associated with blood lipids than other indices, but ABSI showed fewest significant associations to blood lipids, only significantly associated with LDL cholesterol (*R*^2^ = 8.2, *p* = 0.03), total cholesterol (*R*^2^ = 8.8, *p* = 0.03), triglycerides (*R*^2^ = 5.6, *p* = 0.01), and ApoB (*R*^2^ = 8.7, *p* = 0.02).

In the HDP groups, we found positive associations between body mass composition indices and LDL cholesterol, total cholesterol, triglycerides, and ApoB, and negative associations between body mass composition indices and HDL cholesterol and ApoA1, similarly to the control group. Univariable regression analyses are displayed in Supplemental Tables S12 and S13, and beta values and confidence intervals for multivariable regression analyses are displayed in Supplemental Tables S14 and S15. For both the previous HDP groups, the pattern of significant associations between body mass composition indices and blood lipids also remained mostly the same across uni- and multivariable regression analyses. The previous PE group displayed a higher number of significant associations between body mass composition indices and blood lipids than the previous GH group, in multivariable regression analyses (Tables 3 and 4). For the previous PE group, only BMI, WC, and ICO showed significant associations with all blood lipids at postpartum follow-up. The remaining body composition indices significantly associated with most blood lipids, but the patterns of associations differed between body composition indices (Table 3). In the GH group, neither HC nor ABSI showed significant associations with blood lipids. For the remaining body mass composition indices in the GH group, we found significant associations with total cholesterol, LDL cholesterol, and ApoB (Table 4).

**Table 3.** Associations between body mass composition indices and blood lipids for the PE group at follow-up (*n* = 133). Significant associations (*p* < 0.05) are marked with asterix (*).

PE								
	BMI	WC	HC	WHR	WHHR	ICO	ABSI	eTBF
	*R*^2^ (%)	*R*^2^ (%)	*R*^2^ (%)	*R*^2^ (%)	*R*^2^ (%)	*R*^2^ (%)	*R*^2^ (%)	*R*^2^ (%)
LDL	22.18*	22.54*	15.74*	22.81*	21.01*	26.96*	13.83*	24.79*
HDL	31.33*	25.40*	21.27*	16.89*	15.27*	29.78*	7.23	20.80*
Total cholesterol	12.26*	13.67*	9.93	15.98*	14.39*	15.46*	13.10*	15.98*
Triglycerides	28.45*	34.50*	19.62*	25.84*	18.99*	36.59*	10.21*	30.70*
ApoA1	15.52*	11.97*	12.76*	9.56	9.55	13.52*	9.69	10.54
ApoB	25.92*	26.16*	18.38*	23.59*	21.36*	31.04*	13.32*	27.34*

*R*^2^ values for multivariable regression analyses. BMI: body mass index; WC: waist circumference; HC: hip circumference; WHR: waist-to-hip ratio; WHHR: waist-to-hip-to-height ratio; ICO: index of central obesity; ABSI: a body shape index; eTBF: estimated total body fat; LDL: low-density lipoprotein; HDL: high-density lipoprotein; ApoA1: apolipoprotein A1; ApoB: apolipoprotein B; PE: preeclampsia.

**Table 4.** Associations between body mass composition indices and blood lipids for the GH group at follow-up (*n* = 47). Significant associations (*p* < 0.05) are marked with asterix (*).

GH								
	BMI	WC	HC	WHR	WHHR	ICO	ABSI	eTBF
	*R*^2^ (%)	*R*^2^ (%)	*R*^2^ (%)	*R*^2^ (%)	*R*^2^ (%)	*R*^2^ (%)	*R*^2^ (%)	*R*^2^ (%)
LDL	14.88*	14.13*	6.77	11.83*	13.25*	18.27*	1.84	13.90*
HDL	12.17	12.84	11.96	13.02	13.94	13.70	14.05	14.23
Total cholesterol	18.78*	17.14*	11.02	14.05*	13.92*	19.85*	4.91	15.09*
Triglycerides	9.34	8.58	9.87	5.89	5.56	9.38	5.52	8.11
ApoA1	9.33	9.34	9.36	9.33	9.42	9.34	9.34	9.33
ApoB	15.93*	16.67*	6.94	13.81*	18.54*	23.43*	3.30	20.45*

*R*^2^ values for multivariable regression analyses. BMI: body mass index; WC: waist circumference; HC: hip circumference; WHR: waist-to-hip ratio; WHHR: waist-to-hip-to-height ratio; ICO: index of central obesity; ABSI: a body shape index; eTBF: estimated total body fat; LDL: low-density lipoprotein; HDL: high-density lipoprotein; ApoA1: apolipoprotein A1; ApoB: apolipoprotein B; GH: gestational hypertension.

## Discussion

Publications exploring associations between a broad spectrum of body mass composition indices and blood lipids in young women with previous HDP are lacking. A main result of our study is that no body mass composition index appeared superior in association with postpartum dyslipidemia in any of the study groups at 1- to 3-year postpartum follow-up. The associations were heterogenous (Tables 2–4 and Supplemental Tables S6–S15), but importantly, all significant associations showed that a more adverse body composition index was associated with increasing dyslipidemia.

Our findings showed no difference between the different body mass composition indices in their associations with blood lipids at 1 or 3 years postpartum. Ofstad et al. showed stronger associations between nontraditional body composition indices and cardiovascular mortality, as compared to BMI, in a general Norwegian population (HUNT).^17^ This discrepancy may partially be explained by our young study cohort (median age 35), as CVD death is uncommon at low age. Still, Ofstad et al. examined patients aged 19–79 (mean age 48.8 for women), and associations were age-adjusted. Differences in study findings may also be explained by blood lipids used as surrogate marker for CVD, in contrast to cardiovascular mortality in Ofstad et al.’s study.^17^

Our study also showed a more unfavorable metabolic profile (higher body weight, higher BMI and a higher rate of overweight, metabolic syndrome, and hypertension) in women with previous HDP than in controls. The dyslipidemia rate was also higher, although not statistically significant. This unfavorable cardiovascular risk profile, with several risk factor modification opportunities, is well in line with women with previous HDP having an epidemiologically elevated risk for CVD.^35^ Also, the HDP group, in particular the PE group, displayed a significant association between an adverse body composition profile and adverse blood lipid values more often than the control group. This is also in line with more severe HDP forms being at higher risk for CVD.^2^

Multiple studies favor other body mass composition indices over BMI for assessing CVD risk in the general nonpregnant population.^13–21^ In contrast, van Dis et al. found equally strong associations between BMI and WC with CVD risk in a large 10-year cohort study conducted in a general Dutch population,^36^ while Huxley et al.’s review article found conflicting results regarding which body mass composition index is the superior, addressing that most studies evaluating this are cross-sectional.^37^ Our study may underpin this argument in women after pregnancy and childbirth, and longitudinal studies are warranted.

Our study demonstrated more anthropometric measures having significant associations with blood lipids in the previous HDP group, in particular PE, compared with controls. This may indicate a stronger association between overweight and dyslipidemia as a proxy for CVD risk in women after HDP. The previous PE group showed several more significant associations than the GH group, probably indicating a stronger relationship between overweight and CVD risk for women with previous PE than for the control or GH groups. The discrepancy between the PE and GH groups may also be explained by the limited statistical power of the GH group (PE; *n* = 133 versus GH; *n* = 47). We did not subdivide the PE group according to disease onset, as this is not always reliably recorded, but preterm and early-onset PE (<37 and <34 gestational weeks, respectively) are known to confer a higher CVD risk than late-onset PE or GH.^2,38^ We did not compare preterm and term PE subgroups, due to limited power with the reduced sample sizes. However, as indicated from Supplemental Table S4, almost half of the women in the PE group delivered prematurely (median gestational age at delivery was 261 days), suggesting that inclusion of many women with previous early-onset PE may partially explain the stronger relationship between adverse body composition indices and dyslipidemia found in our study.

Despite most women in our cohort being lean (median BMI 23.5 kg/m^2^), and most having a normal BMI (61.1%), other body mass composition indices than BMI could possibly discover specific forms of adverse body mass distribution. Central obesity may be more important than BMI. This is supported by Coutinho et al., showing that patients with coronary artery disease, normal BMI and central obesity have the highest mortality risk, compared with similar patients with other adiposity patterns.^39,40^ Our study groups all had a median WC above reference range (<80 cm),^9^ but no study group differed significantly from controls (Table 1). Our study did not find stronger associations between indices for central obesity and circulating lipids, compared with associations between BMI and circulating lipids. The differing conclusions for central obesity markers from other populations^39,40^ are probably explained by different study endpoints (harder CV endpoints versus lipid surrogate for CVD risk) and younger, healthier study participants (median age 66 years versus 35 years in our study).

Notably, we found fewer significant associations between body mass indices and blood lipids in women with previous uncomplicated pregnancies than in previous pregnancies complicated by HDP. Results remained the same following sensitivity analyses for 1 and 3 years separately (data not shown). Elevated BMI worsens CVD risk factors, including adverse effects on blood pressure, blood sugar, and circulating lipids.^1^ It is therefore possible that overweight and obesity also worsen the adverse effects of HDP on CVD risk. To our knowledge, the mechanisms for this increased vulnerability are unknown. This emphasizes the importance of overweight awareness for young women, and the need for postpartum follow-up, due to HDP being an independent CVD risk factor in addition to overweight and obesity.^2^

We found higher rates of hypertension, metabolic syndrome, and obesity in the previous HDP group, in line with the previously acknowledged overall increased CVD risk in women after HDP.^38,41–43^ The dyslipidemia rates did not differ significantly between the groups. Targeted identification of women with modifiable risk factors for long-term CVD is important. An unfavorable cardiovascular profile, as demonstrated in our and other studies after HDP, is an important argument for assessing long-term CVD risk, especially within the first decade after HDP. Early detection of CVD risk factors, such as following a HDP or GDM pregnancy,^2,41,44^ enables opportunities for early personalized interventions, including lifestyle changes and lowering blood pressure.

### Strengths and limitations

Our study is strengthened by clinically comprehensive and validated pregnancy complication definitions and exclusion of women with chronic diseases that could elevate CVD risk, such as chronic hypertension and diabetes. Breastfeeding is known to impact metabolic profile and reduce CVD risk,^45^ and study participants who breastfed around the time of examination were therefore excluded. Delivering an SGA newborn increases CVD risk independently of other risk factors,^46^ and women who delivered SGA newborns were therefore excluded from the control group. Our study does have some limitations. The cross-sectional nature of this study and the use of surrogate markers (lipids) for hard endpoints (CVD) may be considered as weaknesses. However, hyperlipidemia is strongly associated with CVD,^47^ and we therefore argue that they remain a valid proxy for the risk of hard clinical endpoints. To assess the associations between body mass composition indices and circulating lipids, multiple analyses have been performed. We have not performed corrections for multiple testing due to the increased risk of type I error. As the present paper includes secondary analyses of our observational study outcome data, we did not conduct sample size analyses. Analyses have been conducted for all patients with relevant data available.

Women in our study were mostly of white ethnicity, well- educated, and living in a high-income country. The results are therefore not necessarily generalizable. Epidemiological studies have however shown similar increases in CVD risk after HDP in the Norwegian population as compared to studies in other populations.^38,48^ The low median age of the study cohort also implies a lower CVD risk, compared with the general population. We consider this a clinical advantage, as early CVD risk identification is essential for targeted intervention at an early preclinical disease state with prevention or postponement of CVD and death hopefully.

Our study showed small or nonexisting differences in associations between body composition indices and dyslipidemia. Therefore, implementing a general follow-up after HDP, targeting known CVD risk factors, including lifestyle and overweight assessment, should be a main focus regardless of the choice of body mass composition index. This is important because modifiable CVD risk factors are affected by lifestyle interventions, and women with normal weight may also benefit from this information.^4^ In our postpartum cohort median, BMI was in the upper area of reference values (23.5 kg/m^2^) and 38.9% were overweight or obese. A hypertension rate of 2.7%, prehypertension rate of 10.1%, and a metabolic syndrome rate of 5.7% suggest that lifestyle improvements could confer health improvements and CVD risk reduction, for many women in our assumed clinically healthy cohort. Women with previous PE are more vulnerable for increased circulating lipids and CVD risk with increasing body mass. Whether weight reduction, without other lifestyle changes such as increased physical activity, benefits their metabolic profile remains to be investigated.

American and European guidelines recommend follow-up to evaluate CVD risk after HDP,^27,49–51^ but risk evaluation tools have been found inadequate for this young population.^22^ Women with previous HDP have an elevated risk for developing CVD,^2^ and early awareness and preventing measures are important, even if validated follow-up programs are currently lacking.

## Conclusion

None of the alternative body mass composition indices investigated in our study showed stronger associations with blood lipids than with BMI, at 1- or 3-year postpartum follow-up, supporting the common clinical use of BMI in CVD risk assessment in overweight and obese young women. Women with previous PE showed several more significant associations between body mass composition indices and dyslipidemia than the control and GH groups, but no body mass composition variable appeared superior to others. This may indicate that adiposity in general confers a higher CVD risk in women with previous HDP, especially after PE, than for women with previous uncomplicated pregnancies. Our study supports that all women should be offered postpartum cardiovascular follow-up after HDP. Follow-up should include risk assessment, including regular blood pressure and lipid measurements, together with CVD-reducing lifestyle information. According to our study findings, overweight and obesity evaluation is important for women after HDP, especially PE, to evaluate CVD risk.

## Supplemental Material

sj-docx-1-whe-10.1177_17455057241310316 – Supplemental material for Body mass index is similar to alternative anthropometric indices in evaluating plasma lipids as proxy for cardiovascular disease in women with previous hypertensive disorders of pregnancy: A cross-sectional study Supplemental material, sj-docx-1-whe-10.1177_17455057241310316 for Body mass index is similar to alternative anthropometric indices in evaluating plasma lipids as proxy for cardiovascular disease in women with previous hypertensive disorders of pregnancy: A cross-sectional study by Kristina Klepp, Anne Cathrine Staff, Meryam Sugulle and Kjartan Moe in Women’s Health

sj-docx-2-whe-10.1177_17455057241310316 – Supplemental material for Body mass index is similar to alternative anthropometric indices in evaluating plasma lipids as proxy for cardiovascular disease in women with previous hypertensive disorders of pregnancy: A cross-sectional study Supplemental material, sj-docx-2-whe-10.1177_17455057241310316 for Body mass index is similar to alternative anthropometric indices in evaluating plasma lipids as proxy for cardiovascular disease in women with previous hypertensive disorders of pregnancy: A cross-sectional study by Kristina Klepp, Anne Cathrine Staff, Meryam Sugulle and Kjartan Moe in Women’s Health

## Declarations

**Ethics approval and consent to participate:** The study was approved by the Regional Committee for Medical and Health Research Ethics in South-Eastern Norway (Reference: 2020/23534/ REK sørøst C, 4 September 2020) and conducted in line with the Helsinki Declaration. All women included provided written informed consent.

**Consent for publication:** All women included provided written consent for publication.

**Data availability:** Reasonable requests to access part of the anonymous data set can be sent to corresponding author, but access is restricted because of ongoing clinical follow-up, and the sensitive nature of the data collected for this study. The patient consented to this study and thus obtained ethical body approval.

## References

[1] Roth GA Mensah GA Johnson CO , et al. Global burden of cardiovascular diseases and risk factors, 1990–2019: update from the GBD 2019 study. J Am Coll Cardiol 2020; 76: 2982–3021. 33309175 10.1016/j.jacc.2020.11.010 PMC7755038

[2] Staff AC Redman CW Williams D , et al. Pregnancy and long-term maternal cardiovascular health: progress through harmonization of research cohorts and biobanks. Hypertension 2016; 67: 251–260. 26667417 10.1161/HYPERTENSIONAHA.115.06357

[3] Egeland GM Skurtveit S Staff AC , et al. Pregnancy-related risk factors are associated with a significant burden of treated hypertension within 10 years of delivery: findings from a population-based Norwegian cohort. J Am Heart Assoc 2018; 7: e008318. 10.1161/JAHA.117.008318 PMC6015329 29755036

[4] O’Donnell CJ Elosua R. [Cardiovascular risk factors. Insights from Framingham Heart Study]. Rev Esp Cardiol 2008; 61: 299–310. 18361904

[bibr5-17455057241310316] Yusuf S Hawken S Ounpuu S , et al. Effect of potentially modifiable risk factors associated with myocardial infarction in 52 countries (the INTERHEART study): case-control study. Lancet 2004; 364: 937–952. 15364185 10.1016/S0140-6736(04)17018-9

[6] Egeland GM Klungsøyr K Øyen N , et al. Preconception cardiovascular risk factor differences between gestational hypertension and preeclampsia: cohort Norway study. Hypertension 2016; 67: 1173–1180. 27113053 10.1161/HYPERTENSIONAHA.116.07099 PMC4861703

[7] Whitlock G Lewington S Sherliker P , et al. Body-mass index and cause-specific mortality in 900 000 adults: collaborative analyses of 57 prospective studies. Lancet 2009; 373: 1083–1096. 19299006 10.1016/S0140-6736(09)60318-4 PMC2662372

[8] Aune D Sen A Prasad M , et al. BMI and all cause mortality: systematic review and non-linear dose-response meta-analysis of 230 cohort studies with 3.74 million deaths among 30.3 million participants. BMJ 2016; 353: i2156. 10.1136/bmj.i2156 PMC4856854 27146380

[9] WHO. Waist circumference and waist-hip ratio report of a WHO Expert Consultation. Geneva:w WHO, 2008.

[10] Prentice AM Jebb SA. Beyond body mass index. Obes Rev 2001; 2: 141–147. 12120099 10.1046/j.1467-789x.2001.00031.x

[11] Jackson AS Stanforth PR Gagnon J , et al. The effect of sex, age and race on estimating percentage body fat from body mass index: the Heritage Family Study. Int J Obes Relat Metab Disord 2002; 26: 789–796. 12037649 10.1038/sj.ijo.0802006

[12] Després JP. Body fat distribution and risk of cardiovascular disease: an update. Circulation 2012; 126: 1301–1313. 22949540 10.1161/CIRCULATIONAHA.111.067264

[13] Petursson H Sigurdsson JA Bengtsson C , et al. Body configuration as a predictor of mortality: comparison of five anthropometric measures in a 12 year follow-up of the Norwegian HUNT 2 study. PLoS One 2011; 6: e26621. 10.1371/journal.pone.0026621 PMC3197688 22028926

[bibr14-17455057241310316] Dhana K Kavousi M Ikram MA , et al. Body shape index in comparison with other anthropometric measures in prediction of total and cause-specific mortality. J Epidemiol Community Health 2016; 70: 90–96. 26160362 10.1136/jech-2014-205257

[bibr15-17455057241310316] Schneider HJ Friedrich N Klotsche J , et al. The predictive value of different measures of obesity for incident cardiovascular events and mortality. J Clin Endocrinol Metab 2010; 95: 1777–1785. 20130075 10.1210/jc.2009-1584

[bibr16-17455057241310316] Zhang C Rexrode KM van Dam RM , et al. Abdominal obesity and the risk of all-cause, cardiovascular, and cancer mortality: sixteen years of follow-up in US women. Circulation 2008; 117: 1658–1667. 18362231 10.1161/CIRCULATIONAHA.107.739714

[bibr17-17455057241310316] Ofstad AP Sommer C Birkeland KI , et al. Comparison of the associations between non-traditional and traditional indices of adiposity and cardiovascular mortality: an observational study of one million person-years of follow-up. Int J Obes (Lond) 2019; 43: 1082–1092. 30926954 10.1038/s41366-019-0353-9 PMC6760583

[bibr18-17455057241310316] Song X Jousilahti P Stehouwer CD , et al. Comparison of various surrogate obesity indicators as predictors of cardiovascular mortality in four European populations. Eur J Clin Nutr 2013; 67: 1298–1302. 24149442 10.1038/ejcn.2013.203

[bibr19-17455057241310316] Christakoudi S Tsilidis KK Muller DC , et al. A body shape index (ABSI) achieves better mortality risk stratification than alternative indices of abdominal obesity: results from a large European cohort. Sci Rep 2020; 10: 14541. 32883969 10.1038/s41598-020-71302-5 PMC7471961

[bibr20-17455057241310316] Jayedi A Soltani S Zargar MS , et al. Central fatness and risk of all cause mortality: systematic review and dose-response meta-analysis of 72 prospective cohort studies. BMJ 2020; 370: m3324. 10.1136/bmj.m3324 PMC7509947 32967840

[21] Ashwell M Gunn P Gibson S. Waist-to-height ratio is a better screening tool than waist circumference and BMI for adult cardiometabolic risk factors: systematic review and meta-analysis. Obes Rev 2012; 13: 275–286. 22106927 10.1111/j.1467-789X.2011.00952.x

[22] Moe K Sugulle M Dechend R , et al. Risk prediction of maternal cardiovascular disease one year after hypertensive pregnancy complications or gestational diabetes mellitus. Eur J Prev Cardiol 2020; 27: 1273–1283. 31600083 10.1177/2047487319879791

[23] Piepoli MF Hoes AW Agewall S , et al. 2016 European Guidelines on cardiovascular disease prevention in clinical practice: the Sixth Joint Task Force of the European Society of Cardiology and Other Societies on Cardiovascular Disease Prevention in Clinical Practice (constituted by representatives of 10 societies and by invited experts). Eur Heart J 2016; 37: 2315–2381. 27222591 10.1093/eurheartj/ehw106 PMC4986030

[24] Moe K Sugulle M Dechend R , et al. Functional and structural vascular biomarkers in women 1 year after a hypertensive disorder of pregnancy. Pregnancy Hypertens 2020; 21: 23–29. 32361394 10.1016/j.preghy.2020.04.008

[25] Jacobsen DP Lekva T Moe K , et al. Pregnancy and postpartum levels of circulating maternal sHLA-G in preeclampsia. J Reprod Immunol 2021; 143: 103249. 33254097 10.1016/j.jri.2020.103249

[26] von Elm E Altman DG Egger M , et al. Strengthening the Reporting of Observational Studies in Epidemiology (STROBE) statement: guidelines for reporting observational studies. BMJ 2007; 335: 806–808. 17947786 10.1136/bmj.39335.541782.AD PMC2034723

[27] Brown MA Magee LA Kenny LC , et al. Hypertensive disorders of pregnancy: ISSHP classification, diagnosis, and management recommendations for international practice. Hypertension 2018; 72: 24–43. 29899139 10.1161/HYPERTENSIONAHA.117.10803

[28] Johnsen SL Rasmussen S Wilsgaard T , et al. Longitudinal reference ranges for estimated fetal weight. Acta Obstet Gynecol Scand 2006; 85: 286–297. 16553175 10.1080/00016340600569133

[29] Oslo University Hospital. Laboratoriehåndboken – Medisinsk biokjemi, https://ehandboken.ous-hf.no/folder/1128 (accessed 4 December 2022).

[30] Executive Summary of The Third Report of The National Cholesterol Education Program (NCEP) Expert Panel on Detection, Evaluation, and Treatment of High Blood Cholesterol in Adults (Adult Treatment Panel III). JAMA 2001; 285: 2486–2497. 11368702 10.1001/jama.285.19.2486

[31] WHO. Obesity: preventing and managing the global epidemic. Geneva: WHO, 2000. 11234459

[32] Parikh RM Joshi SR Menon PS , et al. Index of central obesity – a novel parameter. Med Hypotheses 2007; 68: 1272–1275. 17156939 10.1016/j.mehy.2006.10.038

[33] Wilmore JH Behnke AR. An anthropometric estimation of body density and lean body weight in young women. Am J Clin Nutr 1970; 23: 267–274. 5436635 10.1093/ajcn/23.3.267

[34] Krakauer NY Krakauer JC. A new body shape index predicts mortality hazard independently of body mass index. PLoS One 2012; 7: e39504. 10.1371/journal.pone.0039504 PMC3399847 22815707

[35] Yusuf S Joseph P Rangarajan S , et al. Modifiable risk factors, cardiovascular disease, and mortality in 155 722 individuals from 21 high-income, middle-income, and low-income countries (PURE): a prospective cohort study. Lancet 2020; 395: 795–808. 31492503 10.1016/S0140-6736(19)32008-2 PMC8006904

[36] van Dis I Kromhout D Geleijnse JM , et al. Body mass index and waist circumference predict both 10-year nonfatal and fatal cardiovascular disease risk: study conducted in 20,000 Dutch men and women aged 20–65 years. Eur J Cardiovasc Prev Rehabil 2009; 16: 729–734. 19809330 10.1097/HJR.0b013e328331dfc0

[37] Huxley R Mendis S Zheleznyakov E , et al. Body mass index, waist circumference and waist:hip ratio as predictors of cardiovascular risk—a review of the literature. Eur J Clin Nutr 2010; 64: 16–22. 19654593 10.1038/ejcn.2009.68

[38] Bellamy L Casas JP Hingorani AD , et al. Pre-eclampsia and risk of cardiovascular disease and cancer in later life: systematic review and meta-analysis. BMJ 2007; 335: 974. 17975258 10.1136/bmj.39335.385301.BE PMC2072042

[39] Coutinho T Goel K Corrêa de Sá D , et al. Central obesity and survival in subjects with coronary artery disease: a systematic review of the literature and collaborative analysis with individual subject data. J Am Coll Cardiol 2011; 57: 1877–1886. 21545944 10.1016/j.jacc.2010.11.058

[40] Coutinho T Goel K Corrêa de Sá D , et al. Combining body mass index with measures of central obesity in the assessment of mortality in subjects with coronary disease: role of “normal weight central obesity”. J Am Coll Cardiol 2013; 61: 553–560. 23369419 10.1016/j.jacc.2012.10.035

[41] Benschop L Duvekot JJ Roeters van Lennep JE. Future risk of cardiovascular disease risk factors and events in women after a hypertensive disorder of pregnancy. Heart 2019; 105: 1273–1278. 31175138 10.1136/heartjnl-2018-313453 PMC6678044

[bibr42-17455057241310316] Drost JT Arpaci G Ottervanger JP , et al. Cardiovascular risk factors in women 10 years post early preeclampsia: the Preeclampsia Risk EValuation in FEMales study (PREVFEM). Eur J Prev Cardiol 2012; 19: 1138–1144. 21859777 10.1177/1741826711421079

[43] Breetveld NM Ghossein-Doha C van Kuijk S , et al. Cardiovascular disease risk is only elevated in hypertensive, formerly preeclamptic women. BJOG 2015; 122: 1092–1100. 25139045 10.1111/1471-0528.13057

[44] Khosla K Heimberger S Nieman KM , et al. Long-term cardiovascular disease risk in women after hypertensive disorders of pregnancy: recent advances in hypertension. Hypertension 2021; 78: 927–935. 34397272 10.1161/HYPERTENSIONAHA.121.16506 PMC8678921

[45] Schwarz EB Ray RM Stuebe AM , et al. Duration of lactation and risk factors for maternal cardiovascular disease. Obstet Gynecol 2009; 113: 974–982. 19384111 10.1097/01.AOG.0000346884.67796.ca PMC2714700

[46] Bonamy AK Parikh NI Cnattingius S , et al. Birth characteristics and subsequent risks of maternal cardiovascular disease: effects of gestational age and fetal growth. Circulation 2011; 124: 2839–2846. 22124377 10.1161/CIRCULATIONAHA.111.034884

[47] Berberich AJ Hegele RA. A modern approach to dyslipidemia. Endocr Rev 2022; 43: 611–653. 34676866 10.1210/endrev/bnab037 PMC9277652

[48] Irgens HU Reisaeter L Irgens LM , et al. Long term mortality of mothers and fathers after pre-eclampsia: population based cohort study. BMJ 2001; 323: 1213–1217. 11719411 10.1136/bmj.323.7323.1213 PMC59993

[49] Visseren FLJ Mach F Smulders YM , et al. 2021 ESC Guidelines on cardiovascular disease prevention in clinical practice. Eur Heart J 2021; 42: 3227–3337. 34458905 10.1093/eurheartj/ehab484

[bibr50-17455057241310316] Staff AC Kvie A Langsaether E , et al. Hypertensive svangerskapskomplikasjoner og eklampsi (Norwegian), https://www.legeforeningen.no/foreningsledd/fagmed/norsk-gynekologisk-forening/veiledere/veileder-i-fodselshjelp/hypertensive-svangerskapskomplikasjoner-og-eklampsi/ (2020, accessed 3 December 2022).

[51] Arnett DK Blumenthal RS Albert MA , et al. 2019 ACC/AHA Guideline on the Primary Prevention of Cardiovascular Disease: a report of the American College of Cardiology/American Heart Association Task Force on Clinical Practice Guidelines. Circulation 2019; 140: e596–e646. 10.1161/CIR.0000000000000678 PMC7734661 30879355

