# Supplementary material for: Body mass index is similar to alternative anthropometric indices in evaluating plasma lipids as proxy for cardiovascular disease in women with previous hypertensive disorders of pregnancy: A cross-sectional study
Source: Womens Health (Lond). 2025 Jan 28;21:17455057241310316. doi: 10.1177/17455057241310316 (PMC11776011; doi:10.1177/17455057241310316)
Supplement: sj-docx-1-whe-10.1177_17455057241310316 – Supplemental material for Body mass index is similar to alternative anthropometric indices in evaluating plasma lipids as proxy for cardiovascular disease in women with previous hypertensive disorders of pregnancy: A cross-sectional study [file sj-docx-1-whe-10.1177_17455057241310316.docx]

**Supporting Information**

**Supporting Information Figure 1.** Flowchart of inclusion and exclusion to this substudy of the HAPPY study (Health After Pregnancy Complications) one or three years postpartum.

Singleton delivery at Oslo University Hospital - Ullevål 1 or 3 years prior (2013 – 2019)

Index pregnancy with HDP, GDM or SGA ***or*** included in previous study in index pregnancy

Participants approached by the study investigators

Patients declining

Patients excluded:

- Breast feeding
- Pregnant
- Intercurrent disease

n=452

Included in the
HAPPY-study 1or 3 years postpartum (2014-2020)

n=256

Controls: excluded if GDM, SGA-baby or previous PE

Not fasting

n=296

Included to the B-HAPPY substudy (205 at 1 year and 91 at 3 years postpartum)

PE (n=133), GH (n=47) or normal pregnancy (n=116)

Body mass composition indices and blood lipid data available

**Supporting Information Table S1.** Characteristics of the study population, one or three years postpartum (n=296), by index pregnancy group. Data are presented as medians (range) for continuous variables, and as rates (%) (n) for categorical variables. P-values are compared to controls. P-values <0.05 are marked with asterix (*).

|  | Total  (n=296) | Controls  (n= 116) | HDP  (n=180) | PE  (n=133) | GH  (n=47) |
| --- | --- | --- | --- | --- | --- |
| **Fasting glucose (mmol/L)** | 4.7 (3.3 – 6.3) | 4.7 (3.3 – 5.6) | 4.7 (3.7 – 6.3) | 4.7 (3.7 - 6.3) | 4.7 (4.1 – 5.7) |
| **Glucose 2 hours after OGTT (mmol/L)** | 4.7 (2.3 – 10.7) | 4.7 (2.3 – 7.7) | 4.7 (3.1 – 10.7) | 4.7 (3.1 – 10.7) | 4.8 (3.2 – 6.4) |
| **HbA1c (%)** | 5.0 (4.3 – 5.8) | 5.0 (4.5 – 5.5) | 5.0 (4.3 -5.8) | 5.0 (4.5 – 5.8) | 4.9 (4.3 – 5.3) |
| **Premature CVD in 1^st^ degree relative** | 24.8% (73) | 14.7% (17) | 31.1% (56)* | 32.3% (43)* | 27.7% (13) |
| **Education > high school** | 69.9% (207) | 60.3% (70) | 76.1% (137) | 73.7% (98) | 83.0 % (39) |
| **White/not stated ethnicity** | 82.8 % (245) | 79.3% (92) | 85.0% (153)* | 86.5% (109) | 93.6% (44)* |
| **Smoking (Yes)** | 8.1% (24) | 11.2% (13) | 6.1% (11) | 5.3% (7) | 8.5 % (4) |
| **1 year visit data** | 69.3% (205) | 73.3% (85) | 66.7% (120) | 63.2% (84) | 76.6 % (36) |

PE: preeclampsia; GH: gestational hypertension; HDP: hypertensive disorders of pregnancy (PE or GH); OGTT: oral glucose tolerance test; CVD: cardiovascular disease.

**Supporting Information Table S2.** Fasting (≥6 hours) morning serum blood samples at follow up, reference range from department of Clinical Biochemistry, Oslo University Hospital ^1^.

| **Serum blood analysis** | **Reference range** |
| --- | --- |
| Total cholesterol | 3.3-6.9 mmol/l |
| Triglycerides | 0.45-2.60 mmol/l |
| LDL cholesterol | 1.9-4.8 mmol/l |
| HDL cholesterol | 1.0-2.7 mmol/l |
| Lipoprotein ApoA1 | 1.1-2.3 g/l |
| Lipoprotein ApoB | 0.5-1.3 g/l |
| Fasting glucose | 4.0-6.0 mmol/ |
| Glucose 2 hours after OGTT | $\leq7.8$ mmol/l |
| HbA1c | 4.0-6.0 % |

LDL: low density lipoprotein; HDL: high density lipoprotein; ApoA1: apolipoprotein A1; ApoB: apolipoprotein B; OGTT: oral glucose tolerance test.

**Supporting Information Table S3.** An overview and definition of body composition variables used in the present study.

| **Index of body mass composition** | **Equation/unit** |
| --- | --- |
| Body mass index (BMI) ^2^ | Weight [kg]/(height [m])^2^ |
| Waist circumference (WC) ^3^ | Waist circumference [cm] |
| Hip circumference (HC) ^3^ | Hip circumference [cm] |
| Waist-to-hip ratio (WHR) ^3^ | Waist circumference [cm]/hip circumference [cm] |
| Waist-to-height ratio (WHtR) = Index of  central obesity (ICO) ^4^ | Waist circumference [cm]/height [cm] |
| Waist-to-hip-to-height ratio (WHHR) ^5^ | Waist circumference [m]/hip circumference [m]/height [m] (m^-1^) |
| A body shape index (ABSI) ^6^ | WC [m]/(BMI^2/3^ × height^1/2^ [m]) (m^11/6^ kg^-2/3^) |
| Estimated total body fat (eTBF)  (Sex specific formula) ^7^ | Female: 100 × (−76.76 + [4.15 × WC (in.)] – [0.082 × weight (lbs)])/weight) (%) |

**Supporting Information Figure S2.** Cubic splines for associations between BMI and blood lipids with 95% confidence intervals, adjusted for age and smoking for the total cohort at follow-up (n=296).

**
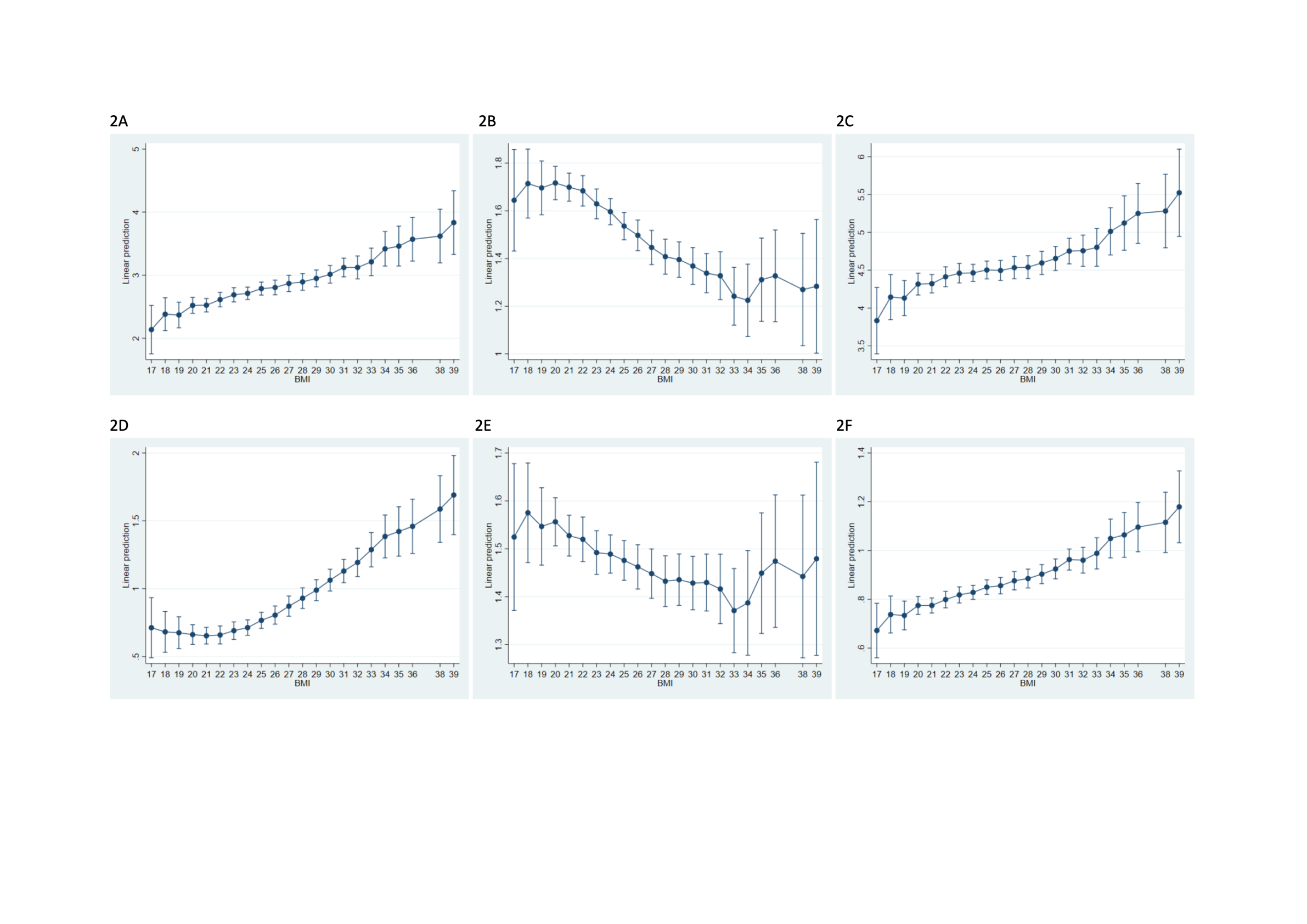
**

1. LDL cholesterol, (B) HDL cholesterol, (C) Total cholesterol, (D) Triglycerides, (E) Apolipoprotein A1, (F) Apolipoprotein B.

**Supporting Information Figure S3.** Cubic splines for associations between WC and blood lipids with 95% confidence intervals, adjusted for age and smoking for the total cohort at follow-up (n=296).


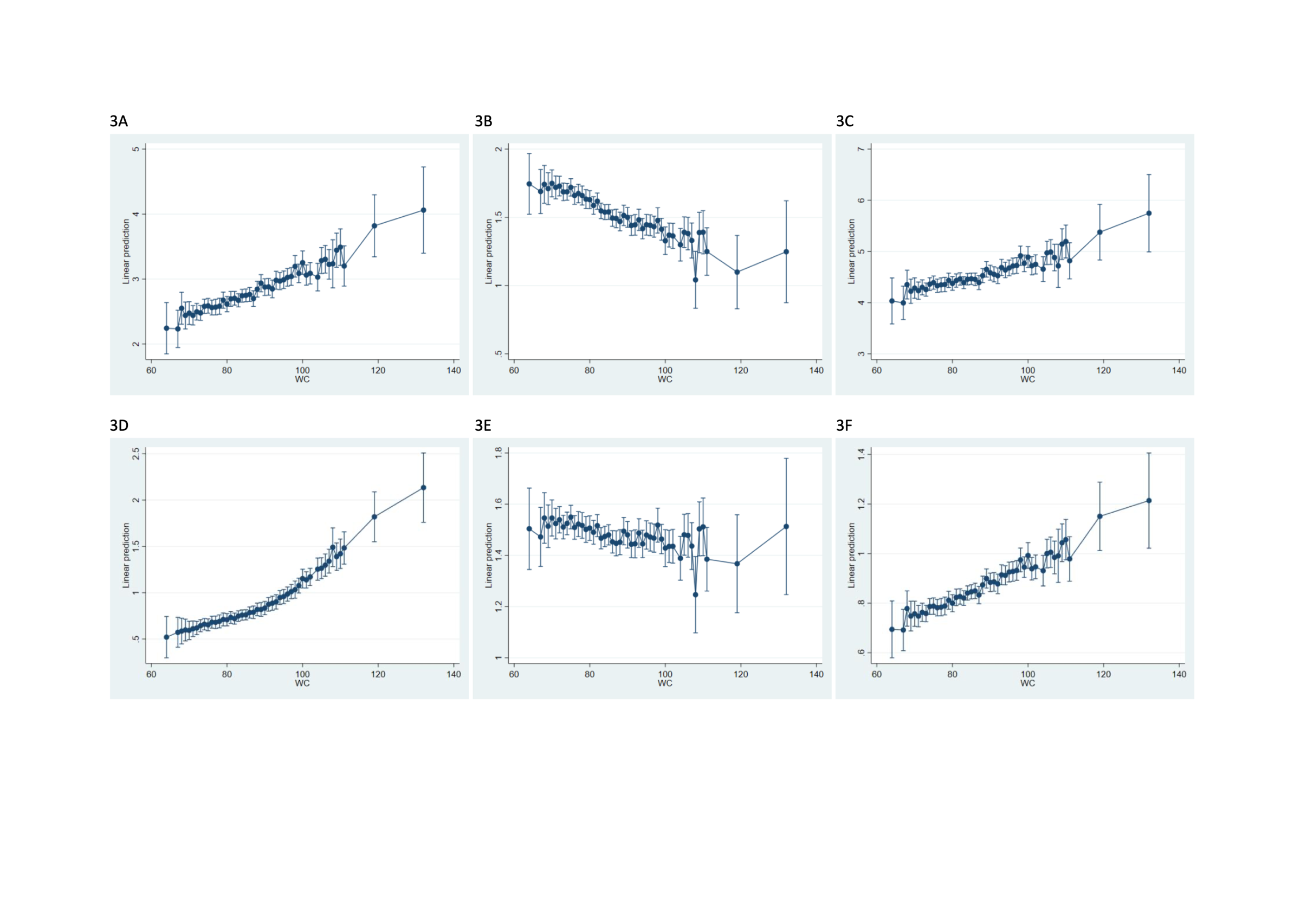


1. LDL cholesterol, (B) HDL cholesterol, (C) Total cholesterol, (D) Triglycerides, (E) Apolipoprotein A1, (F) Apolipoprotein B.

**Supporting Information Figure S4.** Cubic splines for associations between HC and blood lipids with 95% confidence intervals, adjusted for age and smoking for the total cohort at follow-up (n=296).


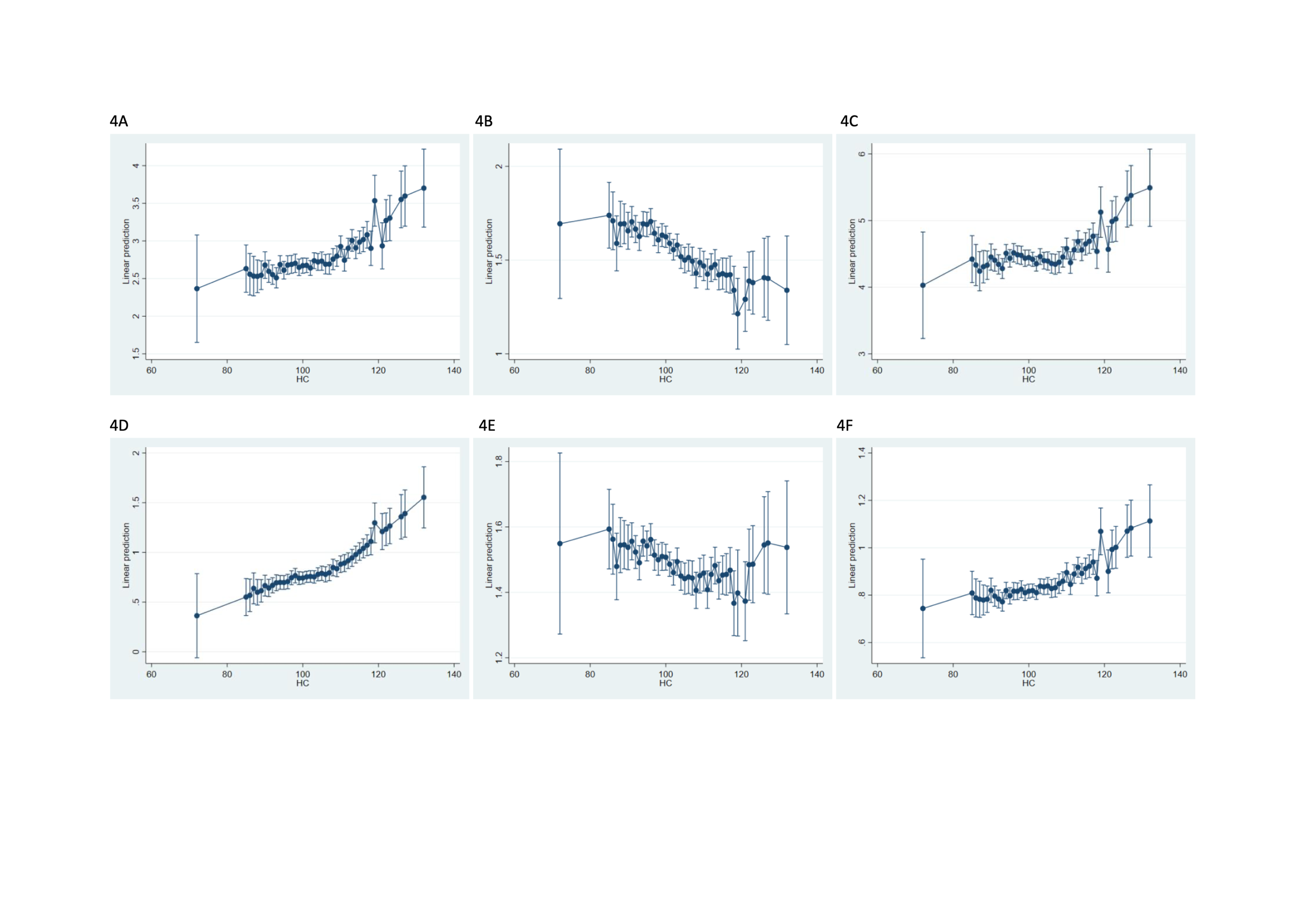


1. LDL cholesterol, (B) HDL cholesterol, (C) Total cholesterol, (D) Triglycerides, (E) Apolipoprotein A1, (F) Apolipoprotein B.

**Supporting Information Figure S5.** Cubic splines for associations between WHR and blood lipids with 95% confidence intervals, adjusted for age and smoking for the total cohort at follow-up (n=296).


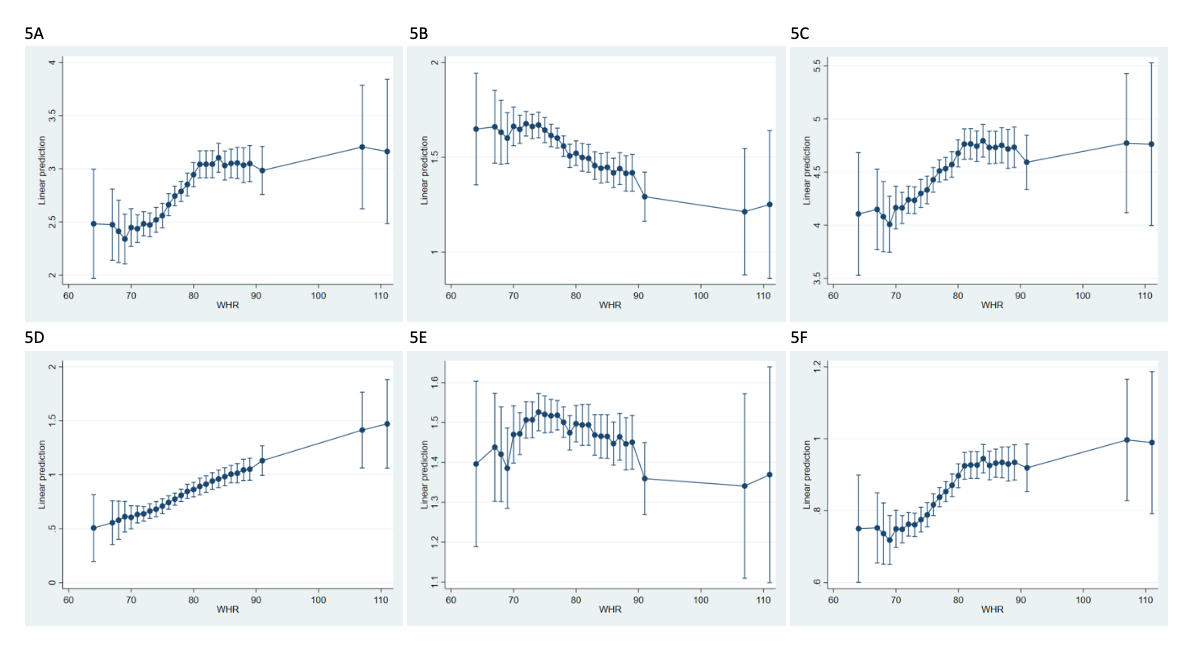


1. LDL cholesterol, (B) HDL cholesterol, (C) Total cholesterol, (D) Triglycerides, (E) Apolipoprotein A1, (F) Apolipoprotein B.

**Supporting Information Figure S6.** Cubic splines for associations between WHHR and blood lipids with 95% confidence intervals, adjusted for age and smoking for the total cohort at follow-up (n=296).


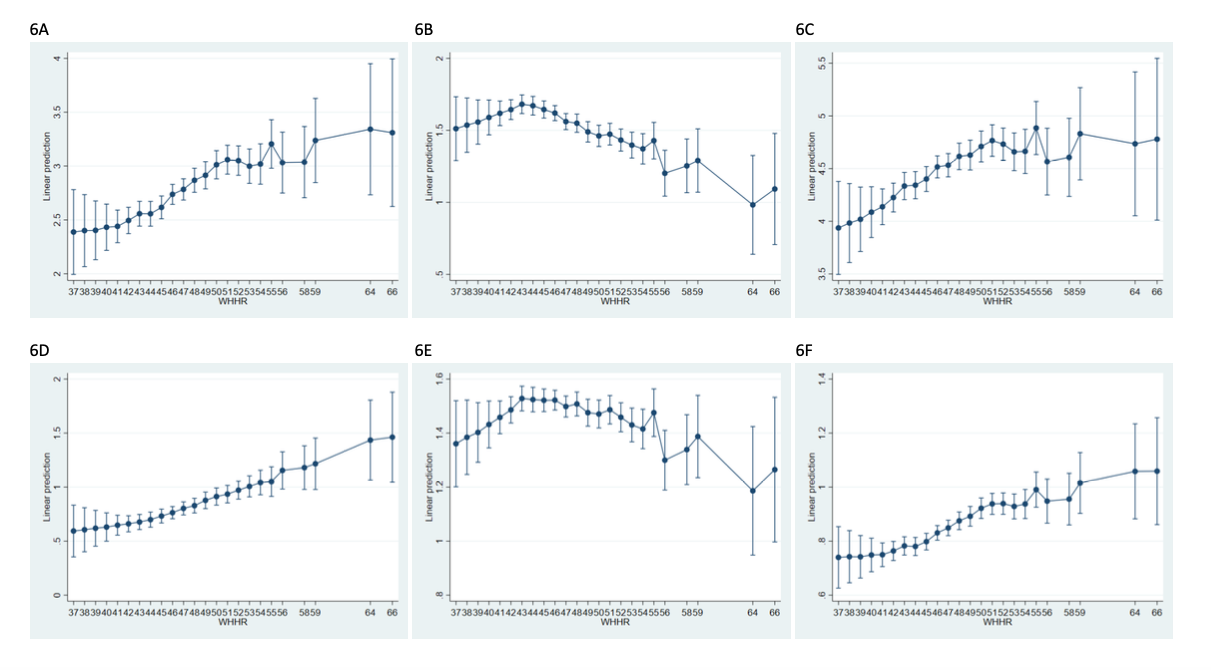


1. LDL cholesterol, (B) HDL cholesterol, (C) Total cholesterol, (D) Triglycerides, (E) Apolipoprotein A1, (F) Apolipoprotein B.

**Supporting Information Figure S7.** Cubic splines for associations between ICO and blood lipids with 95% confidence intervals, adjusted for age and smoking for the total cohort at follow-up (n=296).


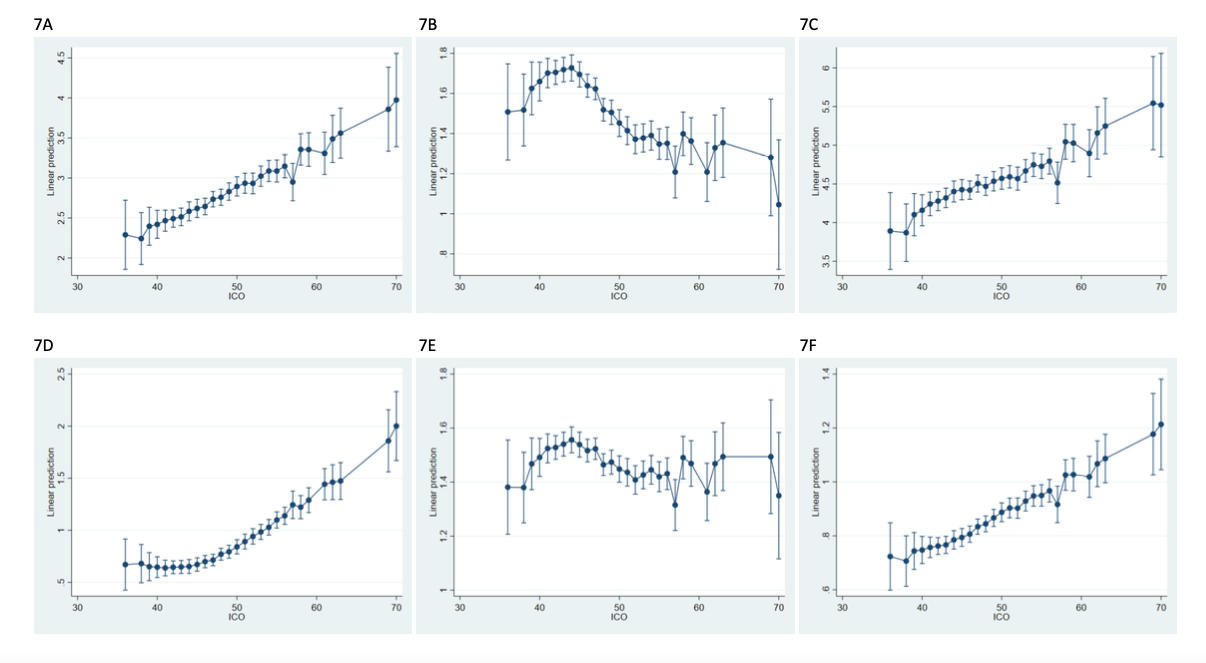


1. LDL cholesterol, (B) HDL cholesterol, (C) Total cholesterol, (D) Triglycerides, (E) Apolipoprotein A1, (F) Apolipoprotein B.

**Supporting Information Figure S8.** Cubic splines for associations between ABSI and blood lipids with 95% confidence intervals, adjusted for age and smoking for the total cohort at follow-up (n=296).


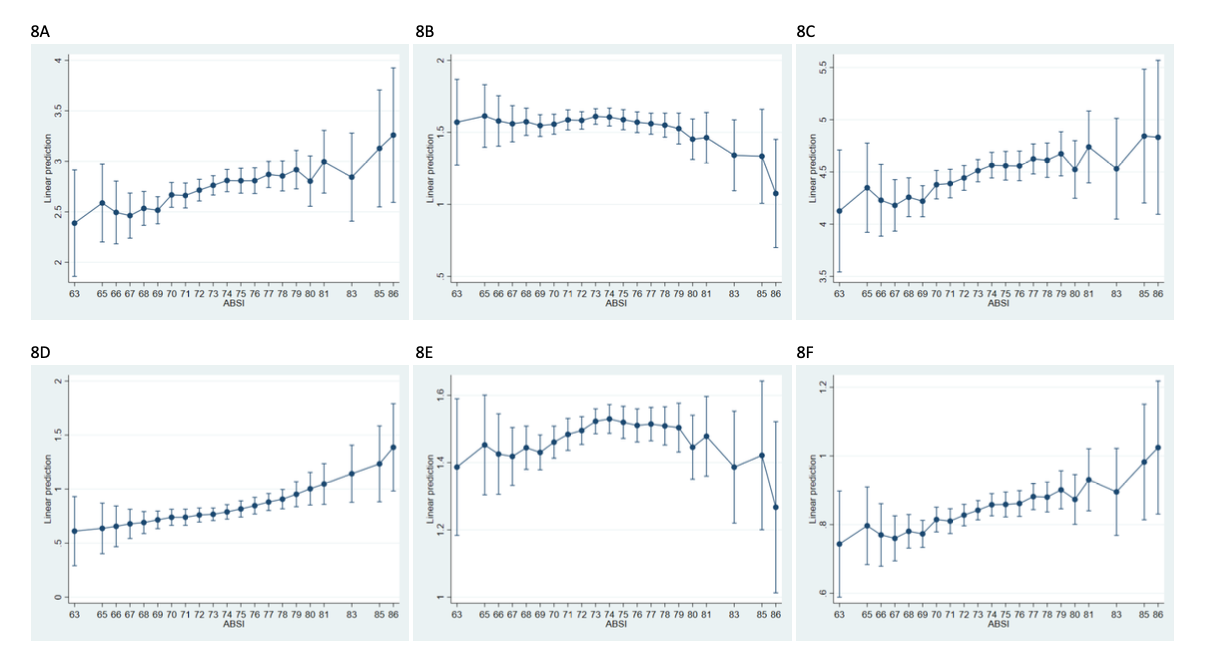


1. LDL cholesterol, (B) HDL cholesterol, (C) Total cholesterol, (D) Triglycerides, (E) Apolipoprotein A1, (F) Apolipoprotein B.

**Supporting Information Figure S9.** Cubic splines for associations between eTBF and blood lipids with 95% confidence intervals, adjusted for age and smoking for the total cohort at follow-up (n=296).


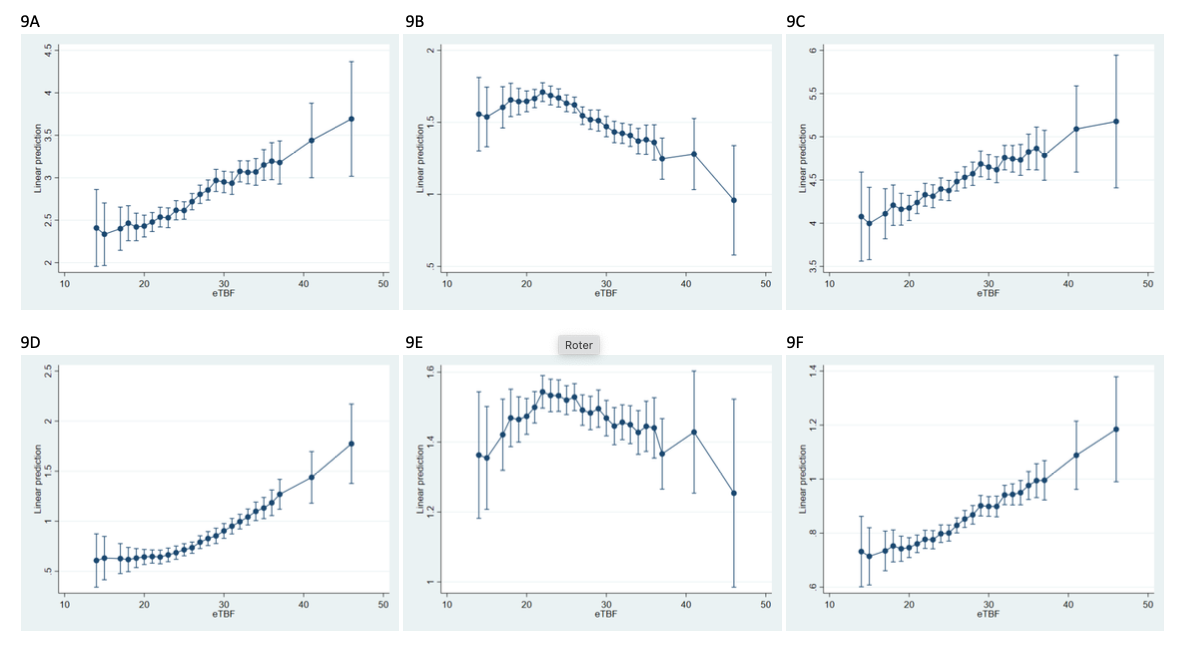


1. LDL cholesterol, (B) HDL cholesterol, (C) Total cholesterol, (D) Triglycerides, (E) Apolipoprotein A1, (F) Apolipoprotein B.

**Supporting Information Table S4.** Clinical pregnancy characteristics of the study population (n=296), by index pregnancy group. Data are presented as medians (range) for continuous variables, and as rates (%) for categorical variables. P-values are compared to controls.

|  | **Total  (n=296)** | **Controls  (n= 116)** | **HDP  (n=180)** | **PE  (n=133)** | **GH  (n=47)** |
| --- | --- | --- | --- | --- | --- |
| **Age at delivery (years)** | 33 (22-45) | 34 (22 - 45) | 33 (22 - 44) | 34 (23 - 44) | 33 (22 - 44) |
| **Weight prepregnancy (kg)** | 65 (43-115) | 63 (44 - 96) | 65 (43 -115) | 65 (43 - 115) | 67 (50 - 101) |
| **Height (cm)** | 167 (150 - 184.5) | 167 (152.5 - 184.5) | 167.5 (150 - 182) | 167 (150 - 182) | 168 (162 - 179) |
| **BMI prepregnancy (kg/m^2^)** | 22.8 (17.3 - 35.5) | 22.4 (17.4 - 35.5) | 23.4 (17.3 - 34.0)* | 23.4 (17.3 - 34.0)* | 23.4 (18.4 - 33.6) |
| **Overweight (BMI ≥ 25 kg/m^2^)** | 27.5% | 18.1% | 25.0% | 25.6 % | 23.4% |
| **Systolic BP week 20 (mmHg)** | 112 (86 - 140) | 110 (90 - 134) | 116 (86 - 140)* | 114 (86 - 140)* | 120 (100 -140)* |
| **Diastolic BP week 20 (mmHg)** | 70 (40 - 100) | 66 (40 - 88) | 73 (52-100)* | 72 (52 - 100)* | 78 (60 - 90)* |
| **Gestational age at delivery (days)** | 273 (177 - 296) | 275 (261 - 296) | 266 (177 - 293)* | 261 (177 - 291)* | 277 (229 - 293) |
| **Gestational age at delivery (weeks)** | 39.0 (25.3 - 42.3) | 39.3 (37.3 - 42.3) | 38.0 (25.3 - 41.9)* | 37.3 (25.3 - 41.6)* | 39.6 (32.7 - 41.9) |
| **Birthweight (g)** | 3259 (510 - 4673) | 3543 (2617 - 4652) | 2844 (510 - 4673)* | 2605 (510 - 4673)* | 3458 (1820 - 4450) |
| **SGA (< 3rd percentile)** | 15.5% | 0.0 % | 25.6%* | 34.6 %* | 0.0 % |
| **Primiparous** | 57.4% | 37.9 % | 70.0%* | 74.4%* | 57.5%* |
| **Female offspring** | 53.7% | 57.8% | 51.1% | 51.1% | 51.1% |

PE: preeclampsia; GH: gestational hypertension; HDP: hypertensive disorders of pregnancy (PE or GH); BMI: body mass index; BP: blood pressure; SGA: small for gestational age. Mann–Whitney U test for continuous variables and Fisher’s mid-p corrected test for categorical variables comparing pregnancy complication groups (e.g., preeclampsia) to controls. Values presented as medians (ranges) or rates.
*p-value < 0.050.

**Supporting Information Table S5.** Dyslipidemia according to pregnancy complication group. Data are presented as number of cases (rates).

|  | **Total  (n=296)** | **Control  (n=116)** | **HDP  (n=180)** | **PE  (n=133)** | **GH  (n=47)** |
| --- | --- | --- | --- | --- | --- |
| **Total cholesterol >6.9 mmol/L** | 1 (0.3%) | 0 (0.0%) | 1 (0.6%) | 1 (0.8%) | 0 (0.0%) |
| **Triglycerides >2.60 mmol/L** | 2 (0.7%) | 0 (0.0%) | 2 (1.1%) | 2 (1.5%) | 0 (0.0%) |
| **HDL <1.0 mmol/L** | 8 (2.7%) | 0 (0.0%) | 8 (4.4%) | 5 (3.8%) | 3 (6.4%) |
| **LDL >4.8 mmol/l** | 1 (0.3%) | 0 (0.0%) | 1 (0.6%) | 1 (0.8%) | 0 (0.0%) |
| **ApoA1 <1.1 g/L** | 9 (3.0%) | 4 (3.4%) | 5 (2.8%) | 4 (3.0%) | 1 (2.1%) |
| **ApoB >1.3 g/L** | 11 (3.7%) | 4 (3.4%) | 7 (3.9%) | 6 (4.5%) | 1 (2.1%) |

PE: preeclampsia; GH: gestational hypertension; HDP: hypertensive disorders of pregnancy (PE or GH); LDL: low density lipoprotein; HDL: high density lipoprotein; ApoA1: apolipoprotein A1; ApoB: apolipoprotein B. Rates are presented according to pregnancy complications groups.

**Supporting Information Table S6.** Unadjusted associations between body mass composition indices and blood lipids for the total cohort at follow-up (n=296). Significant associations (p<0.05) are marked with asterix (*).

| **Total** | | | | | | | | | | | | | | | | |
| --- | --- | --- | --- | --- | --- | --- | --- | --- | --- | --- | --- | --- | --- | --- | --- | --- |
|  | **BMI** | | **WC** | | **HC** | | **WHR** | | **WHHR** | | **ICO** | | **ABSI** | | **eTBF** | |
|  | **B** | **R^2^ (%)** | **B** | **R^2^ (%)** | **B** | **R^2^ (%)** | **B** | **R^2^ (%)** | **B** | **R^2^ (%)** | **B** | **R^2^ (%)** | **B** | **R^2^ (%)** | **B** | **R^2^ (%)** |
| **LDL** | 0.06  (0.04 - 0.08) | 13.0* | 0.02  (0.017 - 0.030) | 13.1* | 0.02  (0.01 - 0.03) | 5.7* | 3.77  (2.56 - 4.98) | 11.4* | 534.27  (355.36 - 713.18) | 10.5* | 5.03  (3.77 - 6.28) | 17.4* | 38.44  (15.90 - 60.98) | 3.7* | 0.05  (0.04 - 0.06) | 14.6* |
| **HDL** | -0.03  (-0.04 - -0.02) | 12.5* | -0.012  (-0.015 - -0.008) | 10.5* | -0.011  (-0.016 - -0.006) | 6.0* | -1.52  (-2.21 - -0.84) | 6.2* | -222.70  (-323.26 - -122.13) | 6.1* | -2.36  (-3.07 - -1.65) | 12.6* | -5.95  (-18.53 - 6.64) | 0.3 | -0.021  (-0.029 - -0.013) | 8.4* |
| **Total cholesterol** | 0.05  (0.03 - 0.07) | 6.5* | 0.02  (0.01 - 0.03) | 7.3* | 0.014  (0.004 - 0.024) | 2.5* | 3.38  (2.02 - 4.74) | 7.5* | 472.35  (271.23 - 673.47) | 6.8* | 4.15  (2.71 - 5.60) | 9.8* | 40.63  (15.77 - 65.49) | 3.4* | 0.04  (0.03 - 0.06) | 9.1* |
| **Triglycerides** | 0.04  (0.03 - 0.05) | 19.2* | 0.018  (0.015 - 0.022) | 23.1* | 0.016  (0.010 - 0.021) | 10.8* | 2.37  (1.66 - 3.08) | 12.9* | 304.53  (198.35 - 410.70) | 9.8* | 3.40  (2.68 - 4.12) | 22.8* | 23.62  (10.32 - 36.92) | 4.0* | 0.03  (0.02 - 0.04) | 16.3* |
| **ApoA1** | -0.010  (-0.17 - -0.003) | 2.7* | -0.003  (-0.005 - 0.000) | 1.2 | -0.003  (-0.007 - 0.000) | 1.2 | -0.29  (-0.77 - 0.19) | 0.5 | -51.06  (-121.91 - 19.79) | 0.7 | -0.62  (-1.13 - -0.10) | 1.9 | 4.45  (-4.16 - 13.05) | 0.4 | -0.004  (-0.010 - 0.002) | 0.7 |
| **ApoB** | 0.019  (0.014 - 0.024) | 14.4* | 0.007  (0.005 - 0.009) | 14.8* | 0.006  (0.003 - 0.008) | 6.1* | 1.16  (0.81 - 1.52) | 12.7* | 173.24  (121.36 - 225.13) | 13.0* | 1.57  (1.21 - 1.94) | 20.1* | 12.59  (5.97 - 19.21) | 4.6* | 0.016  (0.012 - 0.020) | 17.7* |

Univariable regression analyses. BMI: body mass index; WC: waist circumference; HC: hip circumference; WHR: waist-to-hip ratio; WHHR: waist-to-hip-to-height ratio; ICO: index of central obesity; ABSI: a body shape Index; eTBF: estimated total body fat; BP: blood pressure; LDL: low density lipoprotein; HDL: high density lipoprotein; ApoA1: apolipoprotein A1; ApoB: apolipoprotein B.
**Supporting Information Table S7.** Associations between body mass composition indices and blood lipids for the total cohort at follow-up (n=296), adjusted for age and smoking. Significant associations (p<0.05) are marked with asterix (*).

| **TOTAL** | | | | | | | | | | | | | | | | |
| --- | --- | --- | --- | --- | --- | --- | --- | --- | --- | --- | --- | --- | --- | --- | --- | --- |
|  | **BMI** | | **WC** | | **HC** | | **WHR** | | **WHHR** | | **ICO** | | **ABSI** | | **eTBF** | |
|  | **B** | **R^2^  (%)** | **B** | **R^2^  (%)** | **B** | **R^2^  (%)** | **B** | **R^2^  (%)** | **B** | **R^2^  (%)** | **B** | **R^2^  (%)** | **B** | **R^2^  (%)** | **B** | **R^2^  (%)** |
| **LDL** | 0.06  (0.04 - 0.07) | 15.9* | 0.02  (0.01 - 0.03) | 16.1* | 0.02  (0.01 - 0.03) | 10.4* | 3.12  (1.88 - 4.37) | 12.9* | 441.65  (257.77 - 625.53) | 12.3* | 4.43  (3.14 - 5.72) | 18.4* | 25.46  (2.77 - 48.14) | 7.1* | 0.04  (0.03 - 0.06) | 15.4* |
| **HDL** | -0.03  (-0.04 - -0.02) | 15.6* | -0.01  (-0.02 - -0.01) | 13.9* | -0.01  (-0.02 - -0.01) | 9.9* | -1.52  (-2.23 - -0.82) | 8.9* | -217.11  (-321.49 - -112.73) | 8.5* | -2.46  (-3.19 - -1.74) | 16.1* | -5.97  (-18.82 - 6.88) | 3.5 | -0.02  (-0.03 - -0.01) | 11.9* |
| **Total cholesterol** | 0.04  (0.02 - 0.06) | 10.1* | 0.02  (0.01 - 0.02) | 10.8* | 0.01  (0.00 - 0.02) | 7.3* | 2.73  (1.33 - 4.13) | 10.0* | 382.52  (176.06 - 588.97) | 9.6* | 3.49  (2.01 - 4.97) | 12.0* | 27.24  (2.13 - 52.35) | 6.9* | 0.04  (0.02 - 0.05) | 10.9* |
| **Triglycerides** | 0.04  (0.03 - 0.06) | 19.8* | 0.02  (0.02 - 0.02) | 23.7* | 0.02  (0.01 - 0.02) | 11.8* | 2.45  (1.71 - 3.19) | 13.4* | 312.35  (200.80 - 423.90) | 10.2* | 3.57  (2.83 - 4.32) | 24.1* | 23.64  (9.85 - 37.43) | 4.6* | 0.03  (0.03 - 0.04) | 17.5* |
| **ApoA1** | -0.01  (-0.02 - -0.00) | 7.2* | -0.00  (-0.01 - -0.00) | 5.8* | -0.00  (-0.01 - -0.00) | 5.9* | -0.33  (-0.83 - 0.16) | 4.8 | -52.58  (-125.64 - 20.48) | 4.9 | -0.71  (-1.24 - -0.19) | 6.6* | 3.31  (-5.46 - 12.08) | 4.4 | -0.01  (-0.01 - 0.00) | 5.3 |
| **ApoB** | 0.02  (0.01 - 0.02) | 16.7* | 0.01  (0.00 - 0.01) | 17.1* | 0.01  (0.00 - 0.01) | 10.3* | 0.96  (0.60 - 1.32) | 13.5* | 144.16  (91.02 - 197.31) | 13.8* | 1.38  (1.01 - 1.75) | 20.1* | 8.53  (1.90 - 15.17) | 7.3* | 0.01  (0.01 - 0.02) | 17.4* |

Multivariable regression analyses. BMI: body mass index; WC: waist circumference; HC: hip circumference; WHR: waist-to-hip ratio; WHHR: waist-to-hip-to-height ratio; ICO: index of central obesity; ABSI: a body shape Index; eTBF: estimated total body fat; BP: blood pressure; LDL: low density lipoprotein; HDL: high density lipoprotein; ApoA1: apolipoprotein A1; ApoB: apolipoprotein B.

**Supporting Information Table S8.** Unadjusted associations between body mass composition indices and blood lipids for the control group at follow-up (n=116). Significant associations (p<0.05) are marked with asterix (*).

| **Control** | | | | | | | | | | | | | | | | | |
| --- | --- | --- | --- | --- | --- | --- | --- | --- | --- | --- | --- | --- | --- | --- | --- | --- | --- |
|  | | **BMI** | | **WC** | | **HC** | | **WHR** | | **WHHR** | | **ICO** | | **ABSI** | | **eTBF** | |
|  | | **B** | **R^2^ (%)** | **B** | **R^2^ (%)** | **B** | **R^2^ (%)** | **B** | **R^2^ (%)** | **B** | **R^2^ (%)** | **B** | **R^2^ (%)** | **B** | **R^2^ (%)** | **B** | **R^2^ (%)** |
| **LDL** | 0.05  (0.03 - 0.08) | | 10.6* | 0.020  (0.010 - 0.030) | 11.6* | 0.02  (0.00 - 0.03) | 5.4* | 2.45  (0.86 - 4.03) | 7.6* | 325.80  (77.55 - 574.05) | 5.6* | 3.76  (1.92 - 5.60) | 12.5* | 34.06  (1.32 - 66.80) | 3.6* | 0.04  (0.02 - 0.06) | 10.7* |
| **HDL** | -0.02  (-0.04 - -0.01) | | 5.8* | -0.008  (-0.014 - -0.002) | 5.5* | -0.007  (-0.015 - 0.001) | 2.5 | -1.01  (-1.97 - -0.05) | 3.6* | -156.46  (-305.44 - -7.58) | 3.7* | -1.60  (-2.73 - -0.47) | 6.5* | -12.25  (-31.91 - 7.42) | 1.3 | -0.016  (-0.028 - -0.004) | 6.0* |
| **Total cholesterol** | 0.05  (0.01 - 0.08) | | 6.3* | 0.02  (0.01 - 0.03) | 7.4* | 0.013  (-0.003 - 0.028) | 2.3 | 2.50  (0.66 - 4.34) | 6.0* | 350.75  (64.64 - 636.86) | 4.9* | 3.54  (1.37 - 5.70) | 8.4* | 36.29  (-1.41 - 73.99) | 3.1 | 0.04  (0.01 - 0.06) | 7.4* |
| **Triglycerides** | 0.03  (0.02 - 0.05) | | 17.1* | 0.014  (0.009 - 0.019) | 23.6* | 0.009  (0.003 - 0.015) | 6.6* | 1.55  (0.80 - 2.30) | 12.8* | 211.58  (93.11 - 330.05) | 9.9* | 2.29  (1.43 - 3.16) | 19.5* | 21.35  (5.55 - 37.15) | 5.9* | 0.02  (0.01 - 0.03) | 14.1* |
| **ApoA1** | -0.006  (-0.018 - 0.007) | | 0.8 | -0.001  (-0.005 - 0.003) | 0.2 | -0.002  (-0.007 - 0.004) | 0.4 | -0.15  (-0.82 - 0.53) | 0.2 | -32.50  (-137.31 - 72.31) | 0.3 | -0.34  (-1.15 - 0.46) | 0.6 | 0.71  (-12.97 - 14.38) | 0.0 | -0.003  (-0.012 - 0.005) | 0.6 |
| **ApoB** | 0.013  (0.004 - 0.022) | | 7.2* | 0.005  (0.002 - 0.008) | 8.7* | 0.003  (-0.001 - 0.007) | 2.3 | 0.80  (0.32 - 1.28) | 8.8* | 121.71  (46.73 - 196.69) | 8.5* | 1.08  (0.51 - 1.65) | 11.3* | 13.01  (3.09 - 22.92) | 5.7* | 0.012  (0.006 - 0.018) | 12.3* |

Univariable regression analyses. BMI: body mass index; WC: waist circumference; HC: hip circumference; WHR: waist-to-hip ratio; WHHR: waist-to-hip-to-height ratio; ICO: index of central obesity; ABSI: a body shape Index; eTBF: estimated total body fat; BP: blood pressure; LDL: low density lipoprotein; HDL: high density lipoprotein; ApoA1: apolipoprotein A1; ApoB: apolipoprotein B.

**Supporting Information Table S9** Associations between body mass composition indices and blood lipids for the control group at follow-up (n=116), adjusted for age and smoking. Significant associations (p<0.05) are marked with asterix (*).

| **CONTROL** | | | | | | | | |
| --- | --- | --- | --- | --- | --- | --- | --- | --- |
|  | **BMI** | **WC** | **HC** | **WHR** | **WHHR** | **ICO** | **ABSI** | **eTBF** |
|  | **B** | **B** | **B** | **B** | **B** | **B** | **B** | **B** |
| **LDL** | 0.04  (0.01 - 0.07)* | 0.02  (0.01 - 0.03)* | 0.01  (0.00 - 0.03)* | 1.51  (-0.08 - 3.10) | 159.44  (-92.34 - 411.20) | 2.57  (0.64 - 4.51)* | 17.27  (-15.03 - 49.58) | 0.02  (0.00 - 0.04)* |
| **HDL** | -0.02  (-0.04 - -0.01)* | -0.01  (-0.02 - -0.00)* | -0.01  (-0.02 - 0.00) | -1.00  (-2.01 - 0.01) | -145.51  (-305.01 - 13.99) | -1.68  (-2.91 - -0.45)* | -11.71  (-32.31 - 8.90) | -0.02  (-0.03 - -0.00)* |
| **Total cholesterol** | 0.03  (-0.00 - 0.07) | 0.01  (0.00 - 0.03)* | 0.01  (-0.01 - 0.02) | 1.57  (-0.30 - 3.44) | 189.70  (-105.02 - 484.42) | 2.36  (0.06 - 4.65)* | 20.47  (-17.35 - 58.29) | 0.02  (-0.00 - 0.05) |
| **Triglycerides** | 0.04  (0.02 - 0.05)* | 0.02  (0.01 - 0.02)* | 0.01  (0.00 - 0.02)* | 1.65  (0.86 - 2.45)* | 225.09  (97.59 - 352.60)* | 2.64  (1.71 - 3.58)* | 23.13  (6.49 - 39.78)* | 0.02  (0.01 - 0.03)* |
| **ApoA1** | -0.01  (-0.02 - 0.01) | -0.00  (-0.01 - 0.00) | -0.00  (-0.01 - 0.00) | -0.17  (-0.89 - 0.54) | -31.61  (-144.01 - 80.80) | -0.40  (-1.28 - 0.48) | 0.52  (-13.87 - 14.91) | -0.00  (-0.01 - 0.01) |
| **ApoB** | 0.01  (-0.00 - 0.02) | 0.00  (0.00 - 0.01)* | 0.00  (-0.00 - 0.01) | 0.47  (-0.00 - 0.95) | 66.28  (-8.74 - 141.29) | 0.63  (0.04 - 1.21)* | 7.09  (-2.54 - 16.72) | 0.01  (0.00 - 0.01)* |

Beta values (95% confidence interval) for multivariable regression analyses. BMI: body mass index; WC: waist circumference; HC: hip circumference; WHR: waist-to-hip ratio; WHHR: waist-to-hip-to-height ratio; ICO: index of central obesity; ABSI: a body shape Index; eTBF: estimated total body fat; BP: blood pressure; LDL: low density lipoprotein; HDL: high density lipoprotein; ApoA1: apolipoprotein A1; ApoB: apolipoprotein B.

**Supporting Information Table S10.** Unadjusted associations between body mass composition indices and blood lipids for the HDP group (PE or GH) at follow-up (n=180). Significant associations (p<0.05) are marked with asterix (*).

| **HDP** | | | | | | | | | | | | | | | | |
| --- | --- | --- | --- | --- | --- | --- | --- | --- | --- | --- | --- | --- | --- | --- | --- | --- |
|  | **BMI** | | **WC** | | **HC** | | **WHR** | | **WHHR** | | **ICO** | | **ABSI** | | **eTBF** | |
|  | **B** | **R^2^ (%)** | **B** | **R^2^ (%)** | **B** | **R^2^ (%)** | **B** | **R^2^ (%)** | **B** | **R^2^ (%)** | **B** | **R^2^ (%)** | **B** | **R^2^ (%)** | **B** | **R^2^ (%)** |
| **LDL** | 0.07  (0.04 - 0.09) | 14.0* | 0.026  (0.016 - 0.035) | 13.9* | 0.02  (0.01 - 0.03) | 5.7* | 5.07  (3.30 - 6.83) | 15.3* | 695.02  (445.41 - 944.62) | 14.5* | 5.82  (4.12 - 7.53) | 20.3* | 45.05  (13.82 - 76.28) | 4.4* | 0.06  (0.04 - 0.08) | 17.6* |
| **HDL** | -0.04  (-0.05 - -0.03) | 16.4* | -0.013  (-0.018 - -0.008) | 13.6* | -0.013  (-0.019 - -0.006) | 8.1* | -2.05  (-3.02 - -1.09) | 9.0* | -275.31  (-411.56 - -139.06) | 8.2* | -2.81  (-3.73 - -1.90) | 17.1* | -4.34  (-21.15 - 12.47) | 0.2 | -0.025  (-0.35 - -0.014) | 10.4* |
| **Total cholesterol** | 0.05  (0.02 - 0.07) | 6.4* | 0.02  (0.01 - 0.03) | 7.2* | 0.015  (0.001 - 0.028) | 2.5* | 4.25  (2.27 - 6.22) | 9.2* | 566.70  (287.18 - 846.22) | 8.3* | 4.54  (2.59 - 6.49) | 10.6* | 46.88  (13.06 - 80.69) | 4.0* | 0.05  (0.03 - 0.07) | 10.5* |
| **Triglycerides** | 0.05  (0.03 - 0.06) | 19.4* | 0.021  (0.015 - 0.026) | 23.1* | 0.019  (0.011 - 0.026) | 12.3* | 3.23  (2.13 - 4.33) | 15.8* | 379.18  (219.80 - 538.56) | 11.0* | 4.05  (3.01 - 5.08) | 25.1* | 29.75  (10.25 - 49.25) | 4.9* | 0.04  (0.03 - 0.05) | 19.4* |
| **ApoA1** | -0.012  (-0.021 - -0.003) | 4.1* | -0.004  (-0.007 - 0.000) | 2.2 | -0.004  (-0.009 - 0.000) | 1.9 | -0.44  (-1.13 - 0.24) | 0.9 | -65.80  (-162.61 - 31.02) | 1.0 | -0.79  (-1.56 - -0.11) | 3.0* | 6.53  (-4.88 - 17.94) | 0.7 | -0.005  (-0.012 - 0.003) | 0.8 |
| **ApoB** | 0.021  (0.014 - 0.028) | 18.1* | 0.008  (0.006 - 0.011) | 18.1* | 0.007  (0.004 - 0.011) | 8.3* | 1.53  (1.03 - 2.04) | 17.2* | 213.52  (142.79 - 284.25) | 16.8* | 1.87  (1.40 - 2.35) | 25.7* | 14.08  (5.11 - 23.05) | 5.2* | 0.019  (0.014 - 0.025) | 22.1* |

Univariable regression analyses. BMI: body mass index; WC: waist circumference; HC: hip circumference; WHR: waist-to-hip ratio; WHHR: waist-to-hip-to-height ratio; ICO: index of central obesity; ABSI: a body shape Index; eTBF: estimated total body fat; BP: blood pressure; LDL: low density lipoprotein; HDL: high density lipoprotein; ApoA1: apolipoprotein A1; ApoB: apolipoprotein B.

**Supporting Information Table S11.** Associations between body mass composition indices and blood lipids for the HDP group (PE or GH) at follow-up (n=180), adjusted for age and smoking. Significant associations (p<0.05) are marked with asterix (*).

| **HDP** | | | | | | | | | | | | | | | | |
| --- | --- | --- | --- | --- | --- | --- | --- | --- | --- | --- | --- | --- | --- | --- | --- | --- |
|  | **BMI** | | **WC** | | **HC** | | **WHR** | | **WHHR** | | **ICO** | | **ABSI** | | **eTBF** | |
|  | **B** | **R^2^ (%)** | **B** | **R^2^ (%)** | **B** | **R^2^ (%)** | **B** | **R^2^ (%)** | **B** | **R^2^ (%)** | **B** | **R^2^ (%)** | **B** | **R^2^ (%)** | **B** | **R^2^ (%)** |
| **LDL** | 0.06  (0.04 - 0.09) | 18.2* | 0.02  (0.02 - 0.03) | 17.9* | 0.02  (0.01 - 0.03) | 11.0* | 4.72  (2.88 - 6.56) | 17.7* | 643.08  (385.95 - 900.21) | 17.2* | 5.48  (3.75 - 7.21) | 22.9* | 35.01  (3.32 - 66.71) | 8.2* | 0.06  (0.04 - 0.08) | 19.7* |
| **HDL** | -0.04  (-0.05 - -0.02) | 23.1* | -0.01  (-0.02 - -0.01) | 20.3* | -0.01  (-0.02 - -0.01) | 16.5* | -1.87  (-2.86 - -0.88) | 14.7* | -249.77  (-387.92 - -111.61) | 14.1* | -2.76  (-3.67 - -1.85) | 23.5* | -2.42  (-19.16 - 14.32) | 7.9 | -0.02  (-0.04 - -0.01) | 17.5* |
| **Total cholesterol** | 0.05  (0.02 - 0.07) | 12.0* | 0.02  (0.01 - 0.03) | 12.7* | 0.01  (0.00 - 0.03) | 8.5* | 4.08  (2.04 - 6.12) | 14.0* | 536.57  (251.92 - 821.23) | 13.2* | 4.25  (2.29 - 6.22) | 15.2* | 38.02  (3.88 - 72.16) | 8.8* | 0.05  (0.02 - 0.07) | 14.4* |
| **Triglycerides** | 0.05  (0.03 - 0.06) | 19.9* | 0.02  (0.02 - 0.03) | 23.5* | 0.02  (0.01 - 0.03) | 13.9* | 3.21  (2.04 - 4.37) | 15.8* | 366.15  (199.50 - 532.80) | 11.2* | 4.01  (2.94 - 5.07) | 25.1* | 27.66  (7.55 - 47.77) | 5.6* | 0.04  (0.03 - 0.05) | 19.6* |
| **ApoA1** | -0.01  (-0.02 - 0.00) | 12.5* | -0.00  (-0.01 - -0.00) | 10.6* | -0.01  (-0.01 - -0.00) | 10.8* | -0.38  (-1.07 - 0.32) | 9.2 | -58.02  (-155.17 - 39.13) | 9.3 | -0.81  (-1.48 - -0.14) | 11.5* | 6.16  (-5.16 - 17.49) | 9.2 | -0.01  (-0.01 - 0.00) | 9.6 |
| **ApoB** | 0.02  (0.01 - 0.03) | 21.6* | 0.01  (0.01 - 0.01) | 21.5* | 0.01  (0.00 - 0.01) | 13.3* | 1.42  (0.90 - 1.95) | 19.1* | 197.45  (124.52 - 270.38) | 19.0* | 1.77  (1.28 - 2.25) | 27.5* | 11.13  (2.02 - 20.23) | 8.7* | 0.02  (0.01 - 0.02) | 23.5* |

Multivariable regression analyses. BMI: body mass index; WC: waist circumference; HC: hip circumference; WHR: waist-to-hip ratio; WHHR: waist-to-hip-to-height ratio; ICO: index of central obesity; ABSI: a body shape Index; eTBF: estimated total body fat; BP: blood pressure; LDL: low density lipoprotein; HDL: high density lipoprotein; ApoA1: apolipoprotein A1; ApoB: apolipoprotein B.

**Supporting Information Table S12.** Unadjusted associations between body mass composition indices and blood lipids for the PE group at follow-up (n=133). Significant associations (p<0.05) are marked with asterix (*).

| **PE** | | | | | | | | | | | | | | | | |
| --- | --- | --- | --- | --- | --- | --- | --- | --- | --- | --- | --- | --- | --- | --- | --- | --- |
|  | **BMI** | | **WC** | | **HC** | | **WHR** | | **WHHR** | | **ICO** | | **ABSI** | | **eTBF** | |
|  | **B** | **R^2^ (%)** | **B** | **R^2^ (%)** | **B** | **R^2^ (%)** | **B** | **R^2^ (%)** | **B** | **R^2^ (%)** | **B** | **R^2^ (%)** | **B** | **R^2^ (%)** | **B** | **R^2^ (%)** |
| **LDL** | 0.07  (0.04 - 0.09) | 14.1* | 0.026  (0.015 - 0.037) | 14.7* | 0.02  (0.01 - 0.03) | 5.4* | 5.59  (3.57 - 7.62) | 18.5* | 725.17  (435.71 - 1014.63) | 15.8* | 5.96  (3.99 - 7.93) | 21.5* | 59.25  (21.75 - 96.75) | 6.9* | 0.07  (0.04 - 0.09) | 20.1* |
| **HDL** | -0.05  (-0.06 - -0.03) | 25.3* | -0.016  (-0.021 - -0.010) | 19.8* | -0.02  (-0.02 - -0.01) | 12.7* | -2.46  (-3.57 - -1.35) | 12.8* | -321.43  (-478.90 - -163.96) | 11.1* | -3.39  (-4.41 - -2.37) | 24.8* | -1.15  (-21.72 - 19.43) | 0.0 | -0.031  (-0.043 - 0.018) | 15.3* |
| **Total cholesterol** | 0.04 (0.01 - 0.07) | 4.7* | 0.02  (0.01 - 0.03) | 6.2* | 0.011  (-0.004 - 0.027) | 1.5 | 4.49  (2.22 – 6.77) | 10.5* | 559.37  (236.45 - 882.28) | 8.23* | 4.19  (1.93 - 6.45) | 9.3* | 64.78  (24.78 - 104.78) | 7.3* | 0.05  (0.03 - 0.08) | 10.7* |
| **Triglycerides** | 0.06  (0.04 - 0.07) | 26.2* | 0.024  (0.018 - 0.030) | 32.7* | 0.020  (0.012 - 0.029) | 15.1* | 4.11  (2.90 - 5.32) | 25.7* | 488.37  (310.79 - 666.12) | 18.4* | 4.81  (3.70 - 5.92) | 36.0* | 40.94  (17.76 - 64.13) | 8.5* | 0.05  (0.04 - 0.07) | 30.2* |
| **ApoA1** | -0.016  (-0.026 - -0.006) | 6.9* | -0.005  (-0.009 - 0.001) | 3.7* | -0.005  (-0.011 - 0.000) | 3.4* | -0.57  (-1.36 - 0.21) | 1.6 | -82.68  (-192.73 - 27.37) | 1.7 | -1.02  (-1.78 - -0.26) | 5.2* | 8.25  (-5.43 - 21.94) | 1.1 | -0.007  (-0.016 - 0.002) | 2.0 |
| **ApoB** | 0.022  (0.014 - 0.030) | 19.0* | 0.009  (0.006 - 0.012) | 19.4* | 0.007  (0.003 - 0.011) | 8.7* | 1.68  (1.10 - 2.26) | 20.2* | 216.76  (133.47 - 300.04) | 16.9* | 1.92  (1.37 - 2.47) | 26.7* | 17.09  (6.16 - 28.02) | 6.9* | 0.021  (0.014 - 0.027) | 23.5* |

Univariable regression analyses. BMI: body mass index; WC: waist circumference; HC: hip circumference; WHR: waist-to-hip ratio; WHHR: waist-to-hip-to-height ratio; ICO: index of central obesity; ABSI: a body shape Index; eTBF: estimated total body fat; BP: blood pressure; LDL: low density lipoprotein; HDL: high density lipoprotein; ApoA1: apolipoprotein A1; ApoB: apolipoprotein B.

**Supporting Information Table S13.** Unadjusted associations between body mass composition indices and blood lipids for the GH group at follow-up (n=47). Significant associations (p<0.05) are marked with asterix (*).

| **GH** | | | | | | | | | | | | | | | | |
| --- | --- | --- | --- | --- | --- | --- | --- | --- | --- | --- | --- | --- | --- | --- | --- | --- |
|  | **BMI** | | **WC** | | **HC** | | **WHR** | | **WHHR** | | **ICO** | | **ABSI** | | **eTBF** | |
|  | **B** | **R^2^ (%)** | **B** | **R^2^ (%)** | **B** | **R^2^ (%)** | **B** | **R^2^ (%)** | **B** | **R^2^ (%)** | **B** | **R^2^ (%)** | **B** | **R^2^ (%)** | **B** | **R^2^ (%)** |
| **LDL** | 0.06  (0.01 - 0.11) | 13.1* | 0.026  (0.006 - 0.046) | 13.1* | 0.02  (-0.00 - 0.05) | 5.8 | 4.21  (0.51 - 7.91) | 10.5* | 627.26  (120.26 - 1134.26) | 12.1* | 5.32  (1.79 - 8.84) | 17.0* | 20.73  (-37.53 - 78.99) | 1.1 | 0.05  (0.01 - 0.08) | 13.3* |
| **HDL** | -0.01  (-0.04 - 0.02) | 1.0 | -0.004  (-0.016 - 0.007) | 1.4 | -0.003  (-0.016 - 0.011) | 0.4 | -0.88  (-2.93 - 1.16) | 1.7 | -137.04  (-419.03 - 144.96) | 2.1 | -1.02  (-3.03 - 1.00) | 2.2 | -11.81  (-42.48 - 18.86) | 1.3 | -0.010  (-0.030 - 0.011) | 2.0 |
| **Total cholesterol** | 0.07  (0.01 - 0.12) | 12.5* | 0.03  (0.01 - 0.05) | 12.0* | 0.023  (-0.005 - 0.051) | 5.9 | 4.32  (0.16 - 8.48) | 8.8* | 615.30  (41.25 - 1189.34) | 9.4* | 5.53  (1.54 - 9.51) | 14.8* | 15.34  (-49.83 - 80.51) | 0.5 | 0.048  (0.008 - 0.090) | 11.2* |
| **Triglycerides** | 0.03  (0.01 - 0.06) | 4.9 | 0.009  (-0.005 - 0.023) | 3.8 | 0.013  (-0.003 - 0.029) | 5.8 | 0.67  (-1.83 - 3.18) | 0.7 | 51.93  (-295.17 - 399.03) | 0.2 | 1.67  (-0.77 - 4.10) | 4.1 | 5.08  (-32.53 - 42.69) | 0.2 | 0.013  (-0.012 - 0.038) | 2.5 |
| **ApoA1** | -0.001  (-0.021 - 0.018) | 0.1 | -0.000  (-0.009 - 0.008) | 0.0 | 0.000  (-0.010 - 0.010) | 0.0 | -0.05  (-1.58 -1.47) | 0.0 | -11.60  (-225.58 - 202.37) | 0.0 | -0.04  (-1.52 - 1.44) | 0.0 | 2.61  (-19.68 - 24.90) | 0.1 | 0.002  (-0.013 - 0.016) | 0.1 |
| **ApoB** | 0.019  (0.005 - 0.032) | 15.0* | 0.008  (0.003 - 0.014) | 16.5* | 0.006  (-0.001 - 0.013) | 6.7 | 1.31  (0.29 - 2.32) | 13.2* | 210.23  (73.62 - 346.84) | 17.9* | 1.71  (0.76 - 2.66) | 23.1* | 9.79  (-6.31 - 25.90) | 3.3 | 0.016  (0.007 -0.026) | 20.8* |

Univariable regression analyses. BMI: body mass index; WC: waist circumference; HC: hip circumference; WHR: waist-to-hip ratio; WHHR: waist-to-hip-to-height ratio; ICO: index of central obesity; ABSI: a body shape Index; eTBF: estimated total body fat; BP: blood pressure; LDL: low density lipoprotein; HDL: high density lipoprotein; ApoA1: apolipoprotein A1; ApoB: apolipoprotein B.

**Supporting Information Table S14.** Associations between body mass composition indices and blood lipids for the PE group at follow-up (n=133), adjusted for age and smoking. Significant associations (p<0.05) are marked with asterix (*).

| **PE** | | | | | | | | |
| --- | --- | --- | --- | --- | --- | --- | --- | --- |
|  | **BMI** | **WC** | **HC** | **WHR** | **WHHR** | **ICO** | **ABSI** | **eTBF** |
|  | **B** | **B** | **B** | **B** | **B** | **B** | **B** | **B** |
| **LDL** | 0.06  (0.03 - 0.09)* | 0.02  (0.01 - 0.03)* | 0.02  (0.01 - 0.03)* | 4.89  (2.77 - 7.01)* | 628.02  (330.79 - 925.24)* | 5.37  (3.41 - 7.33)* | 43.74  (6.05 - 81.43)* | 0.06  (0.04 - 0.08)* |
| **HDL** | -0.05  (-0.06 - -0.03)* | -0.02  (-0.02 - -0.01)* | -0.02  (-0.02 - 0.01)* | -2.28  (-3.45 - -1.12)* | -289.64  (-452.59 - -126.68)* | -3.31  (-4.33 - -2.30)* | 3.35  (-17.36 - 24.05) | -0.03  (-0.04 - -0.02)* |
| **Total cholesterol** | 0.04  (0.01 - 0.07)* | 0.02  (0.01 - 0.03)* | 0.01  (-0.00 - 0.03) | 4.02  (1.66 - 6.38)* | 494.38  (163.71 - 825.05)* | 3.69  (1.44 - 5.94)* | 52.94  (12.94 - 93.39)* | 0.05  (0.02 - 0.07)* |
| **Triglycerides** | 0.05  (0.04 - 0.07)* | 0.02  (0.02 - 0.03)* | 0.02  (0.01 - 0.03)* | 4.03 (2.73 - 5.32)* | 462.85  (275.15 - 650.56)* | 4.68  (3.54 - 5.82)* | 36.12  (12.12 - 60.11)* | 0.05  (0.04 - 0.06)* |
| **ApoA1** | -0.02  (-0.03 - -0.01)* | -0.00  (-0.01 - -0.00)* | -0.01  (-0.01 - -0.00)* | -0.51  (-1.31 - 0.30) | -70.27  (-182.18 - 41.64) | -1.04  (-1.79 - -0.29)* | 9.11  (-4.53 - 22.76) | -0.01  (-0.02 - 0.00) |
| **ApoB** | 0.02  (0.01 - 0.03)* | 0.01  (0.01 - 0.01)* | 0.01  (0.00 - 0.01)* | 1.48  (0.87 - 2.09)* | 188.53  (102.68 - 274.38)* | 1.75  (1.20 - 2.31)* | 12.74  (1.75 - 23.74)* | 0.02  (0.01 - 0.03)* |

Beta values (95% confidence interval) for multivariable regression analyses. BMI: body mass index; WC: waist circumference; HC: hip circumference; WHR: waist-to-hip ratio; WHHR: waist-to-hip-to-height ratio; ICO: index of central obesity; ABSI: a body shape Index; eTBF: estimated total body fat; BP: blood pressure; LDL: low density lipoprotein; HDL: high density lipoprotein; ApoA1: apolipoprotein A1; ApoB: apolipoprotein B.

**Supporting Information Table S15.** Associations between body mass composition indices and blood lipids for the GH group at follow-up (n=47), adjusted for age and smoking. Significant associations (p<0.05) are marked with asterix (*).

| **GH** | | | | | | | | |
| --- | --- | --- | --- | --- | --- | --- | --- | --- |
|  | **BMI** | **WC** | **HC** | **WHR** | **WHHR** | **ICO** | **ABSI** | **eTBF** |
|  | **B** | **B** | **B** | **B** | **B** | **B** | **B** | **B** |
| **LDL** | 0.07  (0.02 - 0.12)* | 0.03  (0.01 - 0.05)* | 0.02  (-0.01 - 0.05) | 4.37  (0.47 - 8.27)* | 639.88  (107.55 - 1172.21)* | 5.50  (1.76 - 9.22)* | 17.11  (-44.34 - 78.55) | 0.05  (0.01 - 0.09)* |
| **HDL** | -0.01  (-0.03 - 0.02) | -0.00  (-0.02 - 0.01) | -0.00  (-0.02 - 0.01) | -0.77  (-2.82 - 1.28) | -140.81  (-421.25 - 139.62) | -0.96  (-2.98 - 1.07) | -15.68  (-46.09 - 14.73) | -0.01  (-0.03 - 0.01) |
| **Total cholesterol** | 0.07  (0.02 - 0.13)* | 0.03  (0.01 - 0.05)* | 0.02  (-0.00 - 0.05) | 4.53  (0.24 - 8.83)* | 618.81  (27.59 - 1210.03)* | 5.73  (1.62 - 9.85)* | 8.03  (-59.39 - 75.46) | 0.05  (0.01 - 0.09)* |
| **Triglycerides** | 0.02  (-0.01 - 0.06) | 0.01  (-0.01 - 0.02) | 0.01  (-0.00 - 0.03) | 0.76  (-1.82 - 3.34) | 79.96  (-275.84 - 435.76) | 1.76  (-0.76 - 4.27) | 8.20  (-30.41 - 46.82) | 0.02  (-0.01 - 0.04) |
| **ApoA1** | 0.00  (-0.02 - 0.02) | -0.00  (-0.01 - 0.01) | 0.00  (-0.01 - 0.01) | -0.04  (-1.59 - 1.52) | -21.67  (-238.19 - 194.85) | -0.06  (-1.58 - 1.46) | -0.74  (-23.51 - 22.03) | -0.00  (-0.02 - 0.02) |
| **ApoB** | 0.02  (0.01 - 0.03)* | 0.01  (0.00 - 0.01)* | 0.01  (-0.00 - 0.01) | 1.32  (0.24 - 2.39)* | 211.64  (68.05 - 355.23)* | 1.73  (0.73 - 2.74)* | 8.49  (-8.63 - 25.61) | 0.02  (0.01 - 0.03)* |

Beta values (95% confidence interval) for multivariable regression analyses. BMI: body mass index; WC: waist circumference; HC: hip circumference; WHR: waist-to-hip ratio; WHHR: waist-to-hip-to-height ratio; ICO: index of central obesity; ABSI: a body shape Index; eTBF: estimated total body fat; BP: blood pressure; LDL: low density lipoprotein; HDL: high density lipoprotein; ApoA1: apolipoprotein A1; ApoB: apolipoprotein B.

**References:**

1. Oslo University Hospital. Laboratoriehåndboken - Medisinsk biokjemi, <https://ehandboken.ous-hf.no/folder/1128> (accessed 04.12.2022).

2. WHO. *Obesity: Preventing and managing the global epidemic*. Report, WHO, Geneva, 2000.

3. WHO. *Waist Circumference and Waist-Hip Ratio Report of a WHO Expert Consultation*. Report, WHO, Geneva, 2008.

4. Parikh RM, Joshi SR, Menon PS and Shah NS. Index of central obesity - A novel parameter. *Med Hypotheses* 2007; 68: 1272-1275. 2006/12/13. DOI: 10.1016/j.mehy.2006.10.038.

5. Song X, Jousilahti P, Stehouwer CD, et al. Comparison of various surrogate obesity indicators as predictors of cardiovascular mortality in four European populations. *Eur J Clin Nutr* 2013; 67: 1298-1302. 2013/10/24. DOI: 10.1038/ejcn.2013.203.

6. Krakauer NY and Krakauer JC. A new body shape index predicts mortality hazard independently of body mass index. *PLoS One* 2012; 7: e39504. 2012/07/21. DOI: 10.1371/journal.pone.0039504.

7. Wilmore JH and Behnke AR. An anthropometric estimation of body density and lean body weight in young women. *Am J Clin Nutr* 1970; 23: 267-274. 1970/03/01. DOI: 10.1093/ajcn/23.3.267.
